# Plant growth promoting rhizobacteria isolated from halophytes and drought-tolerant plants: genomic characterisation and exploration of phyto-beneficial traits

**DOI:** 10.1038/s41598-020-71652-0

**Published:** 2020-09-09

**Authors:** Kleopatra Leontidou, Savvas Genitsaris, Anastasia Papadopoulou, Nathalie Kamou, Irene Bosmali, Theodora Matsi, Panagiotis Madesis, Despoina Vokou, Katerina Karamanoli, Ifigeneia Mellidou

**Affiliations:** 1 Laboratory of Agricultural Chemistry, School of Agriculture, Aristotle University of Thessaloniki, 54124 Thessaloniki, Greece; 2 International Hellenic University, 57001 Thermi, Greece; 3 Department of Ecology, School of Biology, Aristotle University of Thessaloniki, 54124 Thessaloniki, Greece; 4 Institute of Applied Biosciences, CERTH, 57001 Thessaloniki, Greece; 5 Soil Science Laboratory, School of Agriculture, Aristotle University of Thessaloniki, 54124 Thessaloniki, Greece; 6 Institute of Plant Breeding and Genetic Resources, HAO, 57001 Thermi, Thessaloniki, Greece

**Keywords:** Ecology, Microbiology

## Abstract

Plant growth promoting rhizobacteria (PGPR) are able to provide cross-protection against multiple stress factors and facilitate growth of their plant symbionts in many ways. The aim of this study was to isolate and characterize rhizobacterial strains under natural conditions, associated with naturally occurring representatives of wild plant species and a local tomato cultivar, growing in differently stressed Mediterranean ecosystems. A total of 85 morphologically different rhizospheric strains were isolated; twenty-five exhibited multiple in vitro PGP-associated traits, including phosphate solubilization, indole-3-acetic acid production, and 1-aminocyclopropane-1-carboxylate deaminase activity. Whole genome analysis was applied to eight selected strains for their PGP potential and assigned seven strains to Gammaproteobacteria, and one to Bacteroidetes. The genomes harboured numerous genes involved in plant growth promotion and stress regulation. They also support the notion that the presence of gene clusters with potential PGP functions is affirmative but not necessary for a strain to promote plant growth under abiotic stress conditions. The selected strains were further tested for their ability to stimulate growth under stress. This initial screening led to the identification of some strains as potential PGPR for increasing crop production in a sustainable manner.

## Introduction

Environmental stresses can severely injure the majority of plant species and are among the major constraints to plant growth and crop production worldwide^1–3^. Plants can actively engage microorganisms from the surrounding environment, whereas plant rhizosphere is considered as a hot-spot of microbial activity harboring a wide range of bacteria, many of them exerting positive effects on plants performance and stress resilience^4,5^. The identification of rhizospheric bacterial strains with the potential of enhancing plant growth, denoted as Plant Growth Promoting Rhizobacteria (PGPR), that are able to provide cross-protection against multiple stress factors, became a significant scientific breakthrough towards sustainable agriculture^6,7^.

PGPR can facilitate the growth of their plant symbionts in many different ways, among others by supplying atmospheric nitrogen, synthesizing siderophores, producing phytohormones, such as auxins, gibberellins and cytokinins, solubilizing phosphorus (P) and other minerals, or synthesizing stress alleviating enzymes, such as the 1-aminocyclopropane-1-carboxylate (ACC) deaminase, or cell-wall degrading enzymes^8^. Certain bacteria can also enhance availability of micronutrients, stimulate root growth, and alleviate stress damages by modulating plant defense systems^3,9^. Also, PGPR can act indirectly to benefit plant symbionts by activating induced systemic resistance, increasing antibiotic or antifungal effect, affecting quorum sensing, and/or improving the level of plant cellular metabolites^3,4^.

In an effort to examine ‘healthy’ plant microbiomes and the co-evolutionary signature of host-microorganism interactions^10^, a large number of emblematic PGPR model strains have been characterized, unravelling—to a certain extent—the physiological and the molecular basis of their phyto-beneficial traits^11–13^. Rhizospheric communities contain highly diverse microbiomes, with the most representative bacterial phyla being Acidobacteria, Bacteroidetes, Proteobacteria, Actinobacteria, and Firmicutes^14^. Although a plethora of soil bacteria are considered as PGPR, typically possessing more than one beneficial traits, not all bacterial strains from a certain genus and species have identical functions and interactions with plants^5,15^. The systematic analysis of whole genomes using High-Throughput Sequencing technologies can serve as a powerful strategy to identify causal protein-coding sequences (CDS) contributing to PGP functions^16^. Numerous studies have investigated whole genomes of plant-associated microbes isolated from plantation crops^9^, tall fescue^17^, the desert plant *Indigofera argentea*^18^, maize^19^, chickpea^3^, and sugarcane^20^, or from different types of soils^16,21,22^.

The identification of beneficial strains inhabiting salty and arid ecosystems has received considerable attention, due to their potential use as an alternative and sustainable method to help plants cope with harsh environments^17,18^. Plant-associated microbiota from these areas have developed evolutionary adaptations related to more rigorous stress responses of their host plants, in contrast to microbes found in common cultivated land^14^. In Greece, the synergistic effect of high temperatures and the absence of rainfall during summer, the extended coastline where halophytes develop under extreme salt concentrations, the abundance of aromatic plants, and the presence of special ecosystems like those on volcanic islands, represent an invaluable environment to seek for PGPR.

To the best of our knowledge, there is no published work on plant-associated microbiomes under natural conditions and specifically those associated with wild plant species in diversely stressed ecosystems in the Mediterranean environment. The aim of the present study was to isolate and characterize bacterial strains from drought-tolerant and halotolerant plant species grown under harsh conditions using a polyphasic approach. To evaluate their potential effectiveness in promoting growth, all morphologically different bacterial isolates were tested for their in vitro putative PGP traits, including indole-3-acetic acid (IAA) production, ACC deaminase activity, siderophore production, P-solubilisation, and organic acid production. Whole genome analysis of eight selected strains was employed in order to shed light into the genetic base of potential PGP properties implicated in plant stress tolerance. As a further step, the eight selected strains were screened for their in vivo ability to promote growth under salt or drought stress.

## Results

### Soil properties

**Figure 1:**
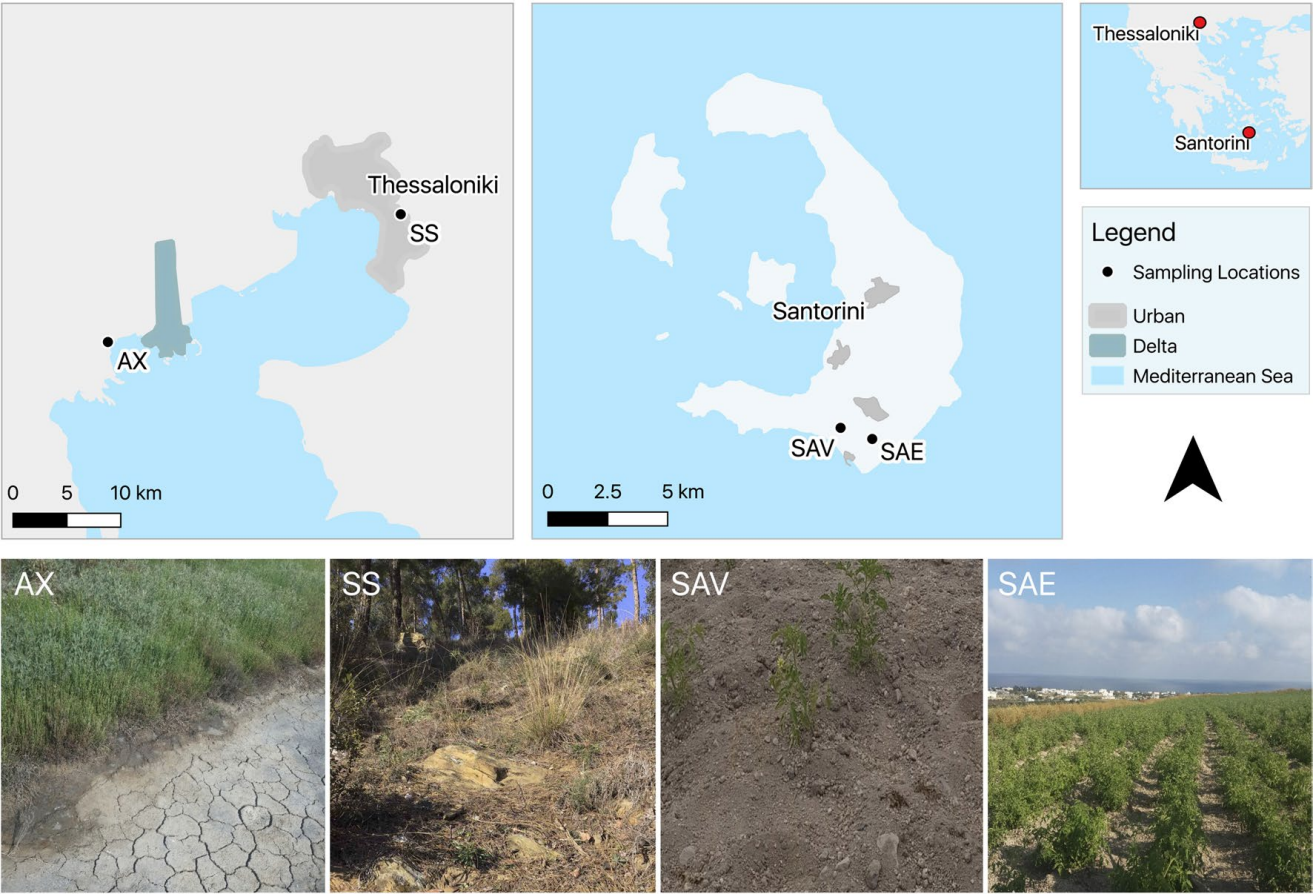
Sampling locations of halophytes from the National Park of Delta Axios (AX, 40°31′19′' N 22°39′02′' E), of aromatic plants from the forest of Seich-Sou (SS, 40°37′41′' N 22°58′15′' E), and of tomato plants from Santorini Island, Vlichada (SAV, 36°20′57′' N 25°25′51′' E) and Emporio (SAE, 36°20′42′' N 25°26′44′' E), as well as and representative photos from these locations.

We sampled soil from the following three areas: (i) the wetland of the National Park of Delta Axios, (ii) the peri-urban forest of Seich Sou, and (iii) Santorini Island in the Aegean Sea, from two sites, Vlichada and Emporio (Fig. 1). The soil from the National Park of Delta Axios primarily consisted of alkaline (pH > 8.0), highly saline-sodic soils (Table 1) with three dominant halophyte species, i.e. *Sarcocornia* sp., *Atriplex* sp., and *Crithmum* sp. Soil salinity around the sampled halophytes ranged from 65 to 72 mS cm^−1^. The forest of Seich-Sou had acidic soils, and it mainly consisted of pine trees, with the understorey containing among others *Cistus* sp., *Thymus* sp., and *Mentha pulegium*. The soils of both areas were coarse in texture and inorganic. However, the acidic soils of Seich-Sou were enriched with organic matter. The soils of the National Park of Delta Axios contained adequate amounts of available NO_3_-N, P and K. The same stands for Seich-Sou soils, except for K, which was far below the sufficiency critical levels, which were 10 mg/kg for NO_3_-N, 10 mg/kg for P and 110 mg/kg for K^23^.

**Table 1:** Chemical properties of the soils collected from the National Park of Delta Axios, the forest of Seich-Sou from where native plants were collected, as well as Santorini island where tomato plants were sampled. Values represent the average of three replicates.

Soil chemical properties	Sampling sites			
	National Park of Delta Axios	Seich-Sou Forest	Santorini Emporio	Santorini Vlichada
pH (1:2 w/v)	8.1	6.6	7.7	8.2
EC (mS cm^−1^)	69	1.0	1.2	0.6
SAR	81	1.1	1.2	0.8
WS K^+^ (meq L^−1^)	0.1	0.2	0.6	0.1
WS Na^+^ (meq L^−1^)	674	1.8	3.0	1.5
WS Ca^++^ (meq L^−1^)	60	3.6	8.5	6.0
WS Mg^++^ (meq L^−1^)	78	1.7	3.4	1.4
CaCO_3_ (%)	2.9	ND	0.8	0.8
OC (%)	1.6	4.7	1.3	0.3
OM (%)	3.3	9.4	2.7	0.6
CEC (cmolc kg^−1^)	12.1	25.5	5.2	2.7
NO_3_-N (mg kg^−1^)	14.2	84.1	57.6	14.3
Olsen P (mg kg^−1^)	16.9	19.0	12.3	2.3
Exch. K^+^ (mg kg^−1^)	965.0	60.0	270.0	58.0

*EC* electrical conductivity, *SAR* sodium adsorption ratio, *WS* water soluble, *OC* organic C, *OM* organic matter, *CEC* cation exchange capacity, *Exch.* Exchangeable, *ND* not detectable.

Soils of Santorini Island, Vlichada and Emporio, were both inorganic, coarse in texture with low cation exchange capacity (CEC), calcareous and alkaline in reaction. However, they represented agricultural ecosystems with quite different soil fertility (Table 1). The Vlichada soil contained available Olsen P and K, at concentrations below the critical sufficiency levels^23^, whereas the opposite was evident for Emporio soil. Conceptually, Vlichada and Emporio were regarded as nutrient-poor and nutrient-rich sites, respectively.

### Morphological and biochemical characterization

A total of 85 rhizobacterial strains were isolated from halophytes and drought-tolerant plants based on their different colony morphology (Table S1). In particular, 28 strains were isolated from halophytes, 30 strains from native plants in the forest of Seich-Sou, and 27 strains from the local tomato cultivar in Santorini Island (six strains from the nutrient-poor Vlichada, and 21 from the nutrient-rich Emporio). Among them, 33 were positive for siderophore production, with the highest production being observed for the strain SSMe03 (Table S1). Bacterial isolates showed significant variation in IAA production in the range of 17.92–112.5 μg mL^−1^. The strain SSCi02 showed the highest IAA production (112.5 μg mL^−1^), while several other strains (SAESo12, SAESo05, SAESo11, SAESo14, SAVSo06) also showed high IAA production.

A total of 25 potential PGPR (Table 2) that were found either positive to both IAA and siderophore production, or to possess very high IAA production, were selected to be screened for additional PGP functions. Four of the 25 selected strains were able to produce alpha-ketobutyrate. These were SAVSo01 and SAVSo04 from the nutrient-poor Vlichada, SAESo21 from the nutrient-rich Emporio, and AXSa06 from the National Park of Delta Axios (Table 2). The highest ACC deaminase activities were observed for the strains SAVSo01 (2,107 nmol ketobutyrate mg protein^−1^ h^−1^), SAVSo04 (853 nmol ketobutyrate mg protein^−1^ h^−1^), AXSa06 (629 nmol ketobutyrate mg protein^−1^ h^−1^) and SAESo19 (222 nmol ketobutyrate mg protein^−1^ h^−1^). A total of eight strains were able to solubilize inorganic P, with the highest P-solubilization indices being recorded for the Seich-Sou strains SSTh11 (6.6 mm) and SSMe04 (4.4 mm). Finally, only two strains (SSCi02, SAESo15) were found positive in organic acid production.

**Table 2:** Biochemical traits of the isolated rhizobacteria, including siderophore production (colony diameter in mm), indole-3-acetic acid (IAA) production (μg mL^−1^ ± SD), 1-aminocyclopropane-1-carboxylate (ACC) deaminase activity (nmol ketobutyrate mg^−1^ protein h^−1^), phosphate solubilization index, and organic acid production (+ , positive; −, negative).

	Strain	Sampling site	Plant species	Siderophore production	IAA production	ACC deaminase activity	Phosphate solubilization index	Organic acids production
**1**	**AXSa06**	**Delta Axios**	***Sarcocornia *sp.**	**5**	**31.25 ± 2.76**	**629**	**1.9**	**−**
**2**	**AXSa07**	**Delta Axios**	***Sarcocornia *sp.**	**6**	**36.88 ± 2.25**	**0**	**0.0**	**−**
3	SSTh03	Seich-Sou Forest	*Thymus* sp.	9	26.88 ± 1.65	0	2.3	**−**
4	SSTh06	Seich-Sou Forest	*Thymus* sp.	8	36.67 ± 2.95	0	2.8	**−**
5	SSTh07	Seich-Sou Forest	*Thymus* sp.	4	32.92 ± 4.52	0	0.0	**−**
**6**	**SSTh08**	**Seich-Sou Forest**	***Thymus *sp.**	**10**	**30.00 ± 1.25**	**0**	**1.8**	**−**
7	SSTh09	Seich-Sou Forest	*Thymus* sp.	6	29.58 ± 4.25	0	0.0	**−**
8	SSTh11	Seich-Sou Forest	*Thymus* sp.	7	22.08 ± 10.5	0	6.6	**−**
9	SSMe01	Seich-Sou Forest	*Mentha pulegium*	4	17.92 ± 7.53	0	0.0	**−**
10	SSMe02	Seich-Sou Forest	*Mentha pulegium*	5	27.29 ± 1.57	0	0.0	**−**
11	SSMe03	Seich-Sou Forest	*Mentha pulegium*	14	34.58 ± 3.61	0	0.0	**−**
12	SSMe04	Seich-Sou Forest	*Mentha pulegium*	9	36.46 ± 4.25	0	4.4	**−**
13	SSCi01	Seich-Sou Forest	*Cistus* sp.	9	25.21 ± 2.01	0	0.0	**−**
**14**	**SSCi02**	**Seich-Sou Forest**	***Cistus *sp.**	**4**	**112.5 ± 2.17**	**0**	**0.0**	** + **
15	SSCi05	Seich-Sou Forest	*Cistus* sp.	11	22.71 ± 0.72	0	0.0	**−**
16	SAVSo01	Vlichada-Santorini	*S.lycopersicum*	4	37.29 ± 4.25	2,107	0.0	**−**
**17**	**SAVSo04**	**Vlichada-Santorini**	***S. lycopersicum***	**5**	**42.50 ± 2.72**	**853**	**2.1**	**−**
18	SAVSo06	Vlichada-Santorini	*S. lycopersicum*	0	64.58 ± 10.6	0	0.0	**−**
19	SAESo05	Emporio-Santorini	*S.lycopersicum*	0	92.71 ± 8.76	0	0.0	**−**
20	SAESo06	Emporio-Santorini	*S.lycopersicum*	6	54.17 ± 6.91	0	0.0	**−**
**21**	**SAESo11**	**Emporio-Santorini**	***S. lycopersicum***	**6**	**90.42 ± 37.8**	**0**	**3.2**	**−**
**22**	**SAESo12**	**Emporio-Santorini**	***S. lycopersicum***	**6**	**105.2 ± 3.66**	**0**	**0.0**	**−**
**23**	**SAESo14**	**Emporio-Santorini**	***S. lycopersicum***	**5**	**74.38 ± 7.81**	**0**	**0.0**	**−**
24	SAESo15	Emporio-Santorini	*S. lycopersicum*	6	47.29 ± 8.06	0	0.0	+
25	SAESo19	Emporio-Santorini	*S.lycopersicum*	6	36.25 ± 3.80	222	0.0	**−**

The strains that were selected for further whole genome sequencing are indicated in bold.

### 16S rRNA gene variability

To characterize taxonomically the 25 morphologically different strains with potential PGP traits, 16S rRNA gene Sanger sequencing was implemented and the strains were affiliated to their closest relative (Table S2). The majority of the strains were closely related to Gammaproteobacteria (68%), followed by Firmicutes (20%), and Bacteroidetes (8%). One 16S rRNA read was affiliated to an unidentified bacterium. The closest relatives belonged to the genera *Pseudomonas* (48%) and *Bacillus* (20%), as well as *Pedobacter*, *Pantoea*, *Luteibacter*, *Acinetobacter*, *Lysobacter*, *Enterobacter* and *Chryseobacterium*.

The combination of results from biochemical (Table 2) and taxonomical (Table S2) characterization of the 25 strains led us to select eight PGPR strains, with diverse PGP functions and from different plant species/ecosystem types, to proceed with whole genome sequencing analysis. In particular, SAESo12 (*Pseudomonas* sp.) and SAESo14 (*Chryseobacterium* sp.) were high IAA producers, SSTh08 (*Pseudomonas* sp.) solubilized P, SAESo11 (*Bacillus* sp.) was high IAA producer and solubilized P, SSCi02 (*Pantoea* sp.) was high IAA producer and organic-acid producer, while AXSa06 (*Bacillus* sp.) and SAVSo04 (*Acinetobacter calcoaceticus*) possessed ACC deaminase activity, produced IAA and solubilized P. All of them were capable of producing detectable amounts of siderophores.

### Taxonomic affiliation

Based on the Prokka functional annotations of the assembled contigs (Table S3) and a set of five conserved reference genes, the phylogenetic tree of the Multilocus Sequence Analysis (MLSA) of these rhizobacterial strains was constructed (Figure S1), grouping the strains into four monophyletic groups, three of them belonging to Gammaproteobacteria, and one belonging to Bacteroidetes. In particular, the strains SSTh08, SAESo11 and SAESo14 were grouped with *Pseudomonas putida*, AXSa06 and AXSa07 with *Pseudomonas oryzihabitans*, SSCi02 was grouped with *Pantoea brenneri*, SAVSo04 with *Acinetobacter calcoaceticus*, and SAESo14 with the Bacteroidetes *Chryseobacterium* sp. (Figure S1). While most of the strains were positioned in agreement with their putative affiliation according to 16S rRNA sequences, the strains AXSa06 and SAESo11, originally affiliated to *Bacillus* sp., were repositioned as *Pseudomonas oryzihabitans* and *Pseudomonas putida*, respectively.

### General genome features

All the eight sequenced genomes contained > 99.5% of high quality reads, with their general genome characteristics being summarized in Table 3. Overall, 3,634,630 pair-end reads were assembled into eight whole genomes, with an average of 454,329 reads per strain. Their estimated genome sizes are consistent with the sizes recorded for their closest relative family members, and varied from 3.7 Mbp (SSCi02) to 5.9 Mbp (SSTh08 and SAESo11). The three genomes that were related to the *Pseudomonas putida* family (SSTh08, SAESo11 and SAESo12) consisted of > 5,300 CDS each, with a total length of > 5.7 Mbp. By contrast, the strain with the lowest number of CDS was SAVSo04 (3,617), classified as *Acinetobacter calcoaceticus*. The genomes related to *Pseudomonas oryzihabitans* family (AXSa06 and AXSa07), contained 4,929 and 4,495 CDS respectively, with a total length of over 4.5 M bp. As for the other three genomes, i.e. SSCi02 (*Pantoea brenneri*), SAVSo04 (*Acinetobacter calcoaceticus*), and SAESo14 (*Chryseobacterium* sp.), they contained 3,712, 3,617, and 4,438 CDS, respectively.

**Table 3:** General features of the whole genomes of the eight rhizobacterial strains that were selected for their Plant-Growth Promoting properties, based on Geneious Prime 2019.2.3 assemblage. The reference genomes were selected based on phylogenetic analyses, shown and described in Figure S1.

Feature	AXSa06	AXSa07	SSTh08	SSCi02	SAVSo04	SAESo11	SAESo12	SAESo14
Phylogenetic affiliation	*Pseudomonas oryzihabitans*	*Pseudomonas oryzihabitans*	*Pseudomonas putida*	*Pantoea brenneri*	*Acinetobacter calcoaceticus*	*Pseudomonas putida*	*Pseudomonas putida*	*Chryseobacterium* sp.
Size (bp)	5,109,344	4,666,115	5,977,297	3,756,065	3,877,365	5,990,360	5,727,198	5,013,127
Assembled reads	534,602	549,284	399,154	429,003	602,990	444,016	279,824	395,757
GC Content (%)	66	65.9	61.7	56.7	39.2	61.7	63.1	36.8
Protein-coding sequences (CDS)	4,929	4,495	5,818	3,712	3,617	5,818	5,352	4,438
rRNA genes	5	15	6	22	18	6	22	21
tRNA genes	58	68	56	77	73	56	73	81
Predicted genes	5,002	4,584	5,884	3,818	3,736	5,884	5,451	4,543
Genes assigned to COGs	2,166	2,208	2,311	2,235	1,693	2,346	2,192	1,574

Within the genomes of the *Pseudomonas* group, a higher number of hypothetical genes and CDS were retrieved in comparison to the strains related to *Pantoea brenneri*, *Acinetobacter calcoaceticus* and *Chryseobacterium* sp. In addition to CDS, several rRNA genes were predicted. The strains of SSCi02, SAESo12 and SAESo14 had over 20 rRNA genes, while AXSa06, SSTh08 and SAESo11 below six. This feature does not seem to be related to the phylogenetic position of the strain, the plant species, or the ecosystems examined. Similarly, tRNA genes varied from 56 (SSTh08 and SAESo11) to 81 (SAESo14).

**Figure 2:**
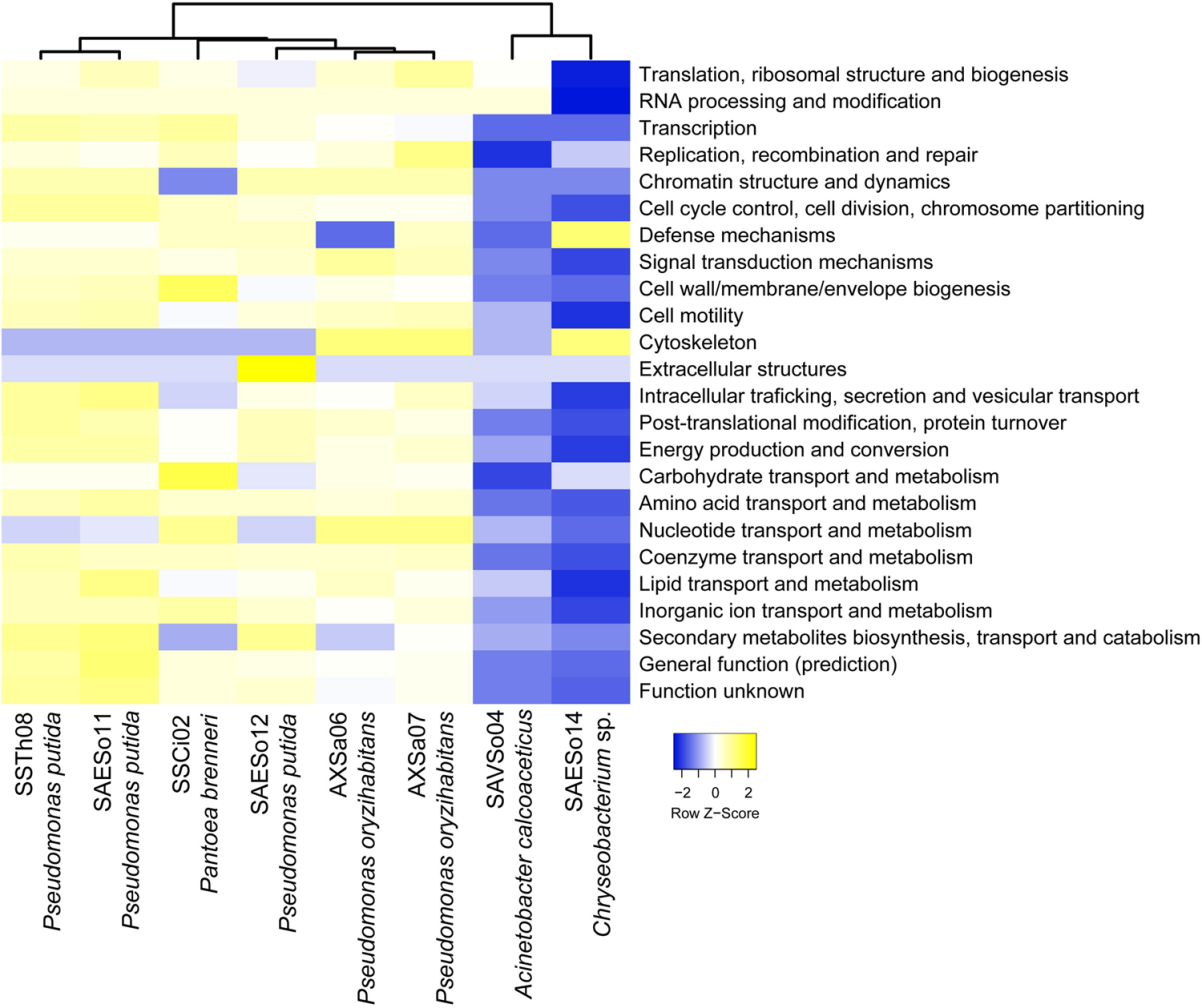
Heatmap using the KEGG BRITE functional classification hits of the contigs assembled from the produced reads of the eight rhizobacterial strains that were selected for their Plant-Growth Promoting properties. Columns are mean-centred, with relative number of genes per functional classification represented by colour (blue: lower numbers; yellow: higher numbers). The Average Linkage clustering method with Euclidean distances was used to cluster the strains according to the similarity of their general functional features.

Classification of the extracted CDS of the eight rhizobacterial strains based on the Clusters of Orthologous Groups of proteins (COG) database demonstrated a close resemblance of the *Pseudomonas-*related strains with *Pantoea brenneri*, similarly to the phylogenetic analysis. The *Acinetobacter calcoaceticus* and the *Chryseobacterium* sp.-related strains were grouped together (Fig. 2), on the basis of their low number of genes per functional classification (Table S4). The COG functional classification of the CDS revealed 1,574 to 2,346 CDS associated with at least one function. Four main functional gene classes related to metabolism were identified in all genomes: (i) Amino acid transportation; (ii) Translation, ribosomal structure and biogenesis; (iii) Energy production and conversion; and (iv) Coenzyme transport and metabolism. In addition, the categories of inorganic ion transport, cell wall and envelope biogenesis, post-translational modification, as well as replication and repair were highly represented in all strains. The genome of the *Chryseobacterium* sp. (SAESo14) included only four potential CDS in the cell-motility category, whereas the other strains, each had > 36 potential genes in this category.

In pairwise comparisons using Mauve alignment, we were able to identify larger conserved segments that appear to be internally free from genome rearrangements, in the case of SAESo12 (*Pseudomonas putida*), with the database reference genome (also *Pseudomonas pudita*), and of SAESo14 identified as *Chryseobacterium s*p. against the reference genome of *Chryseobacterium indologenes* (both isolated from *Zea mays*).

### Genes related to potential PGP traits

**Figure 3:**
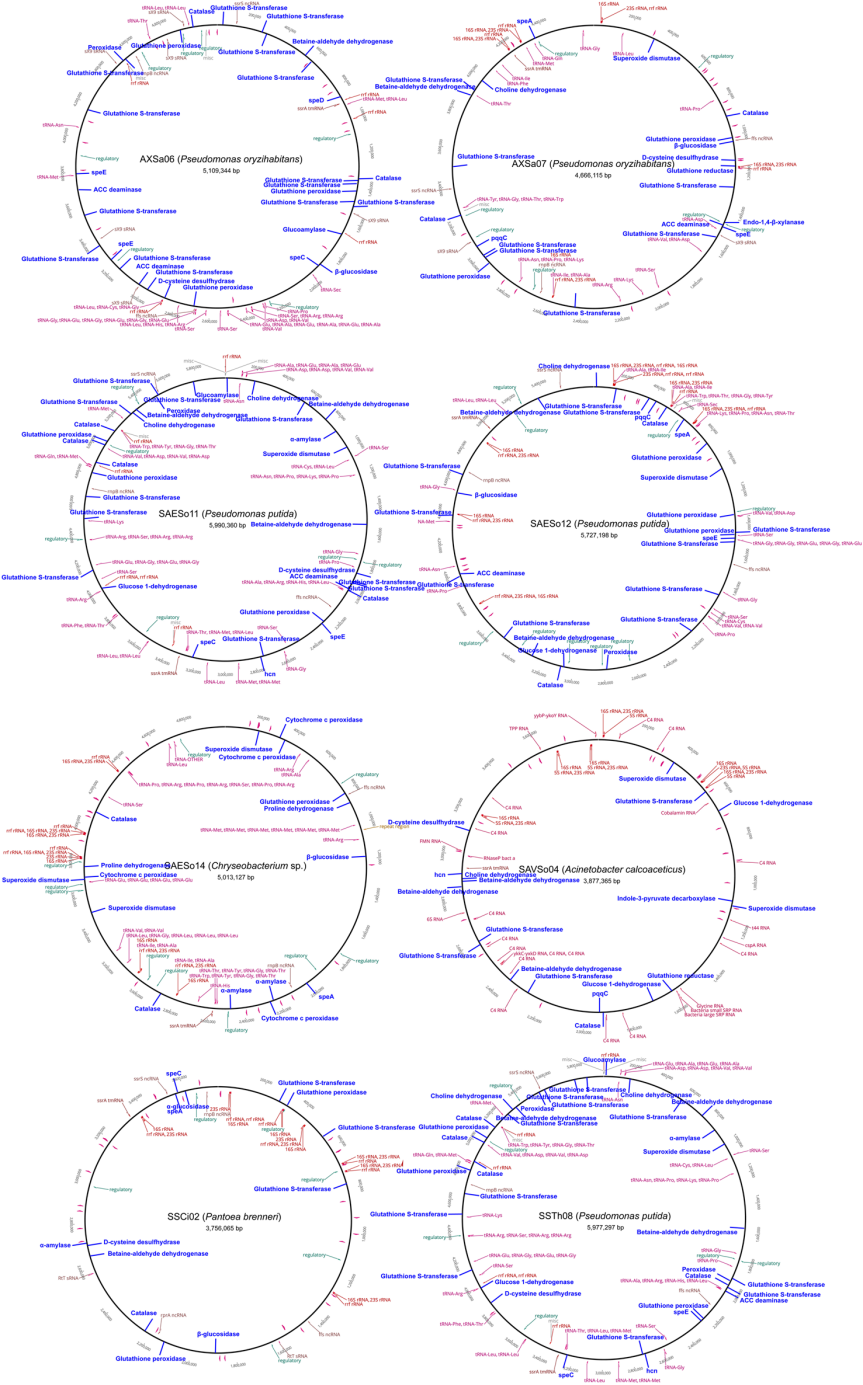
Whole genome representations of the eight rhizobacterial strains that were selected for their Plant-Growth Promoting properties, based on the mapping of the produced reads on reference genomes, using Geneious Prime 2019.2.3. Shades of red indicate RNA coding sequences (CDS); blue lines indicate potential Plant-Growth-Promoting (PGP) CDS; and blue text indicate the respective PGP-related CDS.

The genome data generated in the current study showed that genes involved in stress regulation and plant growth promotion are present in all the sequenced strains (Fig. 3; Table S5). The genomes of AXSa07, SAVSo04 and SAESo12 included genes encoding the pyrrolo-quinolone quinine (*pqq*)-dependent glucose dehydrogenase (*gdh*), while SSCi02, SAVSo04, SAESo11 and SAESo12 carried the cofactor *pqq* gene cluster, which is involved in mineral P-solubilization. Among these genomes, only SAVSo04 and SAESo11 were found to be positive in P-solubilization in vitro (Table 2), suggesting that other mechanisms may co-exist. Neither 2-ketogluconate reductase nor ABC-type phosphate transporters were found at any of the selected genomes (data not shown). Similarly, no nitrogenases or nitrite reductases were detected within the eight genomes (Table S5).

Although IAA production was detected in vitro in all PGPR, only SAVSo04 contained the indole-3-pyruvate decarboxylase (*ipd*) gene. None of these genomes contained tryptophan 2-monooxygenase, aminotransferase and decarboxylase, or indole-3-acetamide hydrolase, whereas all of them comprised other tryptophan synthase (subunit A) orthologs. Multiple genes involved in polyamine biosynthesis were also found in all PGPR, except for SAVSo04.

Most of the selected PGPR contained the *acdS* or the D-cysteine desulfhydrase genes, except for SAESo12 that belongs to *Chryseobacterium* sp. (Fig. 3; Table S5). However, only AXSa06 and SAVSo04 exhibited the enzyme activity in vitro (Table 2).

Furthermore, the whole genomes of the eight PGPR coded for multiple genes involved in antioxidant mechanisms, such as peroxidases (*pox*), catalases (*cat*), superoxide dismutase (*sod*), glutathione peroxidase (*gpx*), reductase (*gr*) and transferase (*gst*) (Table S5). Interestingly, the strains that contained the highest number of gene copies related to oxidative stress alleviation belong to *Pseudomonas* sp. and *Pantoeα brenneri* families. All PGPR genomes coded for hydrolase genes (glucosidases, xylanases and amylases) involved in cell-wall and starch degrading pathways, apart from SAVSo04 (*Acinetobacter* sp.).

Multiple osmoprotectant genes involved in abiotic stress responses were detected within the eight genomes, including choline dehydrogenase, betaine-aldehyde dehydrogenase and proline dehydrogenase (Table S5). The strains with the highest number of such gene copies belong to *Pseudomonas putida*, *Pantoeα brenneri* and *Acinetobacter calcoaceticus* families, isolated from three different collection sites.

### PGPR-elicited growth stimulation under stress

As a further step, the in vivo ability of the selected strains to stimulate plant growth of tomatoes under stress was investigated. Under normal growing conditions, seed inoculation with SAVSo04 and SAESo14 resulted in taller plants, whereas inoculation with SSCi02, SAESo11 and SAESo12 led to smaller plants, compared to non-inoculated ones (Table 4). In the presence of NaCl, there was no significant negative effect in either height or biomass of AXSa07-inoculated plants. By contrast, non-inoculated plants showed a decrease of 20% and 28% in plant height and biomass, respectively, compared to non-stressed plants. The negative effect of drought stress in plants inoculated with SAESo11 and SAESo12 was less profound compared to non-inoculated plants. In particular, under drought stress, plant height showed a 24%, 10% and 11%, decrease in non-inoculated plants, SAESo11-inoculated and SAESo12-inoculated, respectively, compared to non-stressed plants. Accordingly, the above-the-ground biomass reduced 54%, 29%, and 33%. For the other strains, the effect of stress was similar between non-inoculated and inoculated plants.

**Table 4:** Plant height (cm) and total biomass of above the ground part (expressed as total fresh weight g) of tomato seedlings inoculated with the selected potential PGPR exposed to 200 mM NaCl or drought stress for 7 days.

		Plant Height (cm)	Total fresh weight (g)
NB	Control	11.5 ± 0.30a	2.09 ± 0.54a
	200 mM NaCl	8.06 ± 0.85b	1.22 ± 0.26b
AXSa06	Control	11.0 ± 0.70a	2.24 ± 0.41a
	200 mM NaCl	8.41 ± 0.63b	1.27 ± 0.17b
NB	Control	22.4 ± 3.05a	2.66 ± 0.34a
	200 mM NaCl	18.0 ± 2.91b	1.91 ± 0.53b
AXSa07	Control	22.8 ± 4.37a	2.49 ± 0.59a
	200 mM NaCl	20.9 ± 3.17a	2.47 ± 0.51a
NB	Control	27.9 ± 2.25a	3.09 ± 0.57a
	Drought	21.9 ± 1.98b	2.74 ± 0.33b
SSTh08	Control	26.4 ± 0.98a	1.85 ± 0.17a
	Drought	20.8 ± 1.44b	1.69 ± 0.32b
NB	Control	27.9 ± 2.25a	3.09 ± 0.57a
	Drought	22.4 ± 2.71c	2.62 ± 0.46c
SSCi02	Control	26.4 ± 0.98b	1.85 ± 0.17b
	Drought	18.6 ± 2.21d	1.24 ± 0.31d
NB	Control	17.7 ± 1.07b	1.66 ± 0.28b
	Drought	15.8 ± 1.29c	1.33 ± 0.17c
SAVSo04	Control	19.3 ± 1.30a	2.39 ± 0.24a
	Drought	16.9 ± 1.18b	1.40 ± 0.17c
NB	Control	25.9 ± 1.90a	3.78 ± 0.57a
	Drought	19.7 ± 2.28d	1.75 ± 0.55d
SAESo11	Control	23.7 ± 2.18b	3.40 ± 0.54a
	Drought	21.3 ± 2.15c	2.39 ± 0.34b
NB	Control	25.9 ± 1.90a	3.78 ± 0.57a
	Drought	19.7 ± 2.28d	1.75 ± 0.55d
SAESo12	Control	24.1 ± 1.81b	3.32 ± 0.51a
	Drought	21.4 ± 1.84c	2.50 ± 0.36b
NB	Control	17.7 ± 1.07b	1.66 ± 0.28a
	Drought	15.8 ± 1.29c	1.33 ± 0.17c
SAESo14	Control	20.9 ± 1.53a	2.46 ± 0.26b
	Drought	17.5 ± 1.27b	1.23 ± 0.23c

Data are mean values of 20 replicates ± SD.

Different letters indicate significant differences between stressed vs non-stressed plants based on Duncan’s multiple test (*P* < 0.05). For AXSa06, the beginning of stress treatment took place 1 week earlier, i.e. at 14 days after sowing the inoculated seeds.

## Discussion

Plants and bacteria have evolved symbiotic interactions to overcome abiotic stresses^2,3,24–26^. The utilization of PGPR as microbial inoculants is gaining more acceptance as a promising, sustainable and cost-effective strategy of crop improvement^3,26,27^. However, the current application of beneficial microbiota from lab to the field is far from being clearly understood, due to their high complexity under natural ecosystems^7^. Although a broad range of studies from distinct geographical areas have focused on the rhizosphere of stress-tolerant plants^2,28^, to our knowledge, this is the first study addressing the issue of PGPR under natural conditions, associated with wild plant species and landraces, in differently stressed ecosystems of the Mediterranean basin. By examining the rhizospheric bacterial community of the host plants, a total of 85 rhizobacterial strains, with diverse pigmentation and colony morphology, were isolated, including multiples from the same genus. Several of them had orange and red pigmentation due to carotenoids that are formed to help protect bacteria from UV radiation^2^.

Siderophore production and IAA synthesis are related to efficient nutrient uptake and significant enhancement of the root system, respectively^31–33^, especially during stress periods^34^. Siderophore-producing bacteria can indirectly promote plant growth via preventing the proliferation of pathogens by decreasing the amount of available iron^4,8^. Many bacterial strains possessed this trait, with the highest efficiencies being recorded for those isolated from the native species at Seich-Sou forest. Similar to previous studies^35,36^, the majority of the isolated bacteria were able to synthesize IAA, but to a different extent depending on the sampling site. The observed range may be linked to the various pathways for bacterial biosynthesis of IAA^37,38^. The highest efficiencies in IAA production were recorded from the strains isolated from the nutrient-rich soil of Emporio, and the highest IAA producer was a *Pantoea*-related strain, a taxon reported previously for high IAA production^39^. High levels of IAA promote the formation of lateral roots^34^, and the increase root length and surface area^8^, which might be essential for tomato plants grown under no irrigation scheme in the drought-stricken areas of Santorini.

Ethylene has multiple negative roles in plant root growth, while reduced ethylene emission has been correlated with enhanced nutrient and water uptake^34,40,41^. Colonization of roots by these strains may help plants alleviate the ethylene-induced effect on root and shoot development, especially under salt stress^42^, by restoring normal plant development^8^. In this study, only four isolates had in vitro ACC deaminase activity by producing sufficient amount of alpha-ketobutyrate^15^. The two strains possessing the highest efficiency in ACC deaminase were isolated from the nutrient-poor and alkaline areas of Vlichada (Santorini) and Delta Axios, suggesting that bacterial ACC deaminase activity could be predominantly selected by plants growing under harsh soil and environmental stresses^43^. The fact that the positive strains had also relatively low IAA production reveals the complementarity of different PGP functions linked to microbiome diversity at a given environment.

Phosphate-solubilizing bacteria can indirectly enhance plant growth through the more efficient nutrient uptake^44^. Among the P-solubilizers, five of them were isolated from the rhizosphere of native plants grown at the peri-urban Seich-Sou forest. Considering that organic acids enable inorganic P-solubilization in the soil^44^, the presence of strains that can boost this process could be valuable, especially at acidic soils. Indeed, among the two bacterial isolates that could produce organic acids, one was isolated from the forest (acidic soils), where it probably contributes to P-solubilization.

The 16S rRNA taxonomic annotation of 25 strains with potential PGP traits, revealed that the isolates belonged to family members known for their PGP activity, such as *Pseudomonas*, and *Bacillus*^34,45–47^. Also, the rest of the bacterial genera have been associated with plant growth, including *Enterobacter*^46^, *Pantoea*^39^, *Acinetobacter*^48^, *Chryseobacterium*^49^, *Pedobacter*^50^, *Luteibacter*^51^, and *Lysobacter*^52^. Different plant species, having different root architecture and length, also showed significant differences in root microbiome assemblages^10^. Whether a higher relative abundance of Gammaproteobacteria affects plant growth and performance under adverse condition remains rather obscure.

The whole genome analysis of the eight selected PGPR showed that they belonged to five different species: *Pseudomonas oryzihabitans*, *Pseudomonas putida*, *Pantoea brenneri*, *Acinetobacter calcoaceticus* and *Chryseobacterium* sp. Two of the strains were repositioned within Gammaproteobacteria, and were found to be closer to *Pseudomonas oryzihabitans* and *Pseudomonas putida*, rather than their 16S rRNA gene based annotation to *Bacillus* sp. Taxonomy annotations based on genetic markers, such as 16S rRNA gene, are essentially predictions depending on a number of different parameters, such as the reference database and authoritative assignments of environmental sequences^53^; therefore, such a taxonomy deviation is likely. The genome size of all strains was in agreement with previously reported genomes of their related strains in NCBI database. All the strains had a high combined number of rRNAs and tRNA genes, which is a typical characteristic of soil microbes that are able to respond rapidly to changing nutrient availability^54^.

Functional analysis by COGs of the genomes revealed that a high number of characterized CDS were involved in essential functions for bacterial survival, growth and competition with other soil bacteria^55,56^. The COGs distribution of the *Pseudomonas*-related strains and the *Pantoea brenneri*-related strain showed high similarity, suggesting that they have comparable biological niches. Noteworthy, the niche represented by these strains was found in all three study areas, possibly evidencing a model bacterium with representative functions, able to survive, compete and adapt in the harsh conditions of the sampling sites, and play important roles in the symbiont plant’s growth. Indeed, *Pseudomonas* and *Pantoea* strains have been widely recognized as rhizobacteria with potential PGP traits^57–59^. On the other hand, the *Acinetobacter calcoaceticus* and *Chryseobacterium* sp. strains had dissimilar distribution of COG functions in comparison to the *Pseudomonas-Pantoea* cluster. Several reports have also identified *Acinetobacter* and *Chryseobacterium* relatives as potential PGPR^47,60^. Based on MAUVE alignments, large conserved genome segments were only identified for two strains isolated from the tomato cultivars in Emporio, Santorini. These findings may indicate that crop PGP strains of the same species might have relatively well preserved genomes in contrast with bacterial strains of the same species isolated from native plants (i.e. halophytes or aromatic plants).

As P-availability in soil is generally limited, the presence of P-solubilizing bacteria could be essential^16^. Many P-solubilizing microorganisms require external *pqq* for strong P-solubilization in vitro^61^. The *pqq* and *gcd* genes, encoding pyrroquinoline quinone synthase and glucose 1-dehydrogenase, respectively, are both associated with the production of gluconic acid which is involved in inorganic P-solubilization^62^. Several strains had orthologous copies of these genes, including those related to *Pseudomonas* sp., *Pantoea brenneri*, and *Acinetobacter calcoaceticus*. However, among these strains, only SAVSo04 and SAESo11 were able to solubilize P in vitro. In the case of SAVSo04, the association between the very low levels of P in the soil (Vlichada, Santorini), the in vitro ability of strain to solubilize P, and the appearance of P-solubilizing genes within its genome, highlights the importance of such bacteria for plant survival under P-deficient conditions. Although the *pqq* coenzyme is not exclusively linked to P uptake^21^, the co-existence of these genes along with the in vitro evidence described above, support the notion that these two strains are P-solubilizing microorganisms in line with previous studies^61,63^. By contrast, the in vitroP-solubilizing bacteria (AXSa06 and SSTh08) lacked orthologs of the *pqq* or *gds* genes, implying a putative alternative mechanism that requires further elucidation. Other strains had orthologs of the relevant genes but lacked the activity. Considering the fact that bacteria may lose some of their abilities when the corresponding cation is present in adequate amounts, or in case that it is provided by a consortium of microbiota^16^, it is evident that the environment where bacteria are grown is of outmost importance. This explanation may be relevant for SAESo12 (isolated from the P-rich environment of Santorini, Emporio) that possessed the relevant orthologous genes but lacked the activity.

The biosynthesis of IAA in bacteria can be achieved by tryptophan-dependent and tryptophan-independent pathways^16^. Although the majority of strains were able to produce IAA in vitro only one strain, SAVSo04, possessed orthologs of indole-3-pyruvate decarboxylase (*ipdc*), the key gene responsible for the conversion of indole-3-pyruvate to indole-3-acetaldehyde (indole-3-pyruvate pathway). By contrast, the majority of strains had orthologous genes of tryptophan synthase, while other genes including tryptophan 2-monooxygenase (indole-3-acetamide pathway) were absent, suggesting that tryptophan-independent pathways may also be important.

Polyamines (PA) have been also correlated with lateral root, ethylene modulation and oxidative stress alleviation^21,64^. Considering the role of spermidine in bacteria biofilm formation^65^, genes involved in PA pathways may constitute an additional mechanism related to PGP properties, as suggested in *Serratia marcescens*^21^ or *Bacillus megaterium* STB1^66^. The majority of the selected PGPR possessed PA-related genes, except for SAVSo04 whose genome contained the *ipdc* gene. The PA pathway probably acts as an additional PGPR-related mechanism involved in plant growth promotion that requires further investigation.

Microbes possessing ACC deaminase activity can alleviate the detrimental effect of abiotic stress through degrading the ethylene precursor ACC into ammonia and alpha-ketobutyrate^26^. ACC deaminase activity has been detected predominantly in *Pseudomonas* and *Mesorhizobium* strains, but also reported in members of the genus *Enterobacter*^18^. Among the selected PGPR, all of them carried orthologs of *acdS* and D-cysteine desulfhydrase, apart from SAESo14 (*Chysobacterium* sp.). Nevertheless, as indicated above, only two of them (AXSa06 and SAVSo04) exhibited high levels of ACC deaminase activity in vitro It has been previously reported that strains positive for *acdS* gene can be negative for enzyme activity, as in the case of a *Mesorhizobium* strain, that only expresses the activity when it is placed within a root nodule^43^.

Antioxidant enzymes, such as pox, cat, sod, gr and gst play crucial roles in the detoxification of Reactive Oxygen Species (ROS) which are largely produced in stressful environments. A broad number of studies support the PGPR-mediated stress alleviation in different plant species and stress conditions^3,20^. All genomes possessed a significant amount of orthologs encoding antioxidant enzymes, probably reflecting the need for survival under the extreme environments from where they have been isolated.

Some biocontrol bacteria are also able to synthesize a similar panel of hydrolytic enzymes responsible for the breakdown of carbohydrates, such as chitinases, xylanases, amylases and peroxidases^8^. The majority of PGPR genomes contained genes involved in cell-wall and starch degrading pathways, apart from the *Acinetobacter*-related strain. Numerous putative biocontrol agents with hydrolytic activities have been reported, including the genera of *Bacillus*, *Pantoea*, *Enterobacter* and *Pseudomonas* sp.^67^.

Several genes considered as osmoprotectants were found within the eight genomes, with the highest number of copies observed for *Pantoea *sp. and *Pseudomonas putida* families, which also had the highest number of antioxidant-related genes. Both gene families are regulated by transcription factors that can activate adaptive responses to eliminate stress^24^. Furthermore, under drought stress, PGPR have been demonstrated to enhance sugar accumulation in plants enabling the osmoprotection of photosynthetic organs^3^.

Subsequently, preliminary screening of the eight characterize strains had led us to the identification of three strains with significant enhancement of plant growth under stress, compared to non-inoculated plants. No surprise, two of them were isolated from tomato plants grown under adverse conditions, suggesting that PGP bacteria symbiosis is probably more crucial for the survival of cultivated plant species, under unfavourable growing conditions, rather than wild species, which may have evolved highly complicated mechanisms of survival, regardless the surrounding microbiota community. There are multiple reports on the PGP effect of different bacterial strains in a broad number of plant species, clearly evidencing that this symbiotic stimulation is both plant host- and bacterial- species specific^2,3,9,21,24^.

In conclusion, the present study provides important clues related to the genetic and biochemical characterization of potential PGPR, isolated from adverse environments in the Mediterranean basin. The different but complementary genomic and PGP characteristics of the isolated strains highlights the functional convergence of phylogenetically different microbes, and signifies the future research perspectives towards a more holistic approach in the use of beneficial microbiota. The complementarity of their PGP functions will pave the way to their application as consortia of microbes, as an alternative sustainable strategy to improve agricultural production under unfavourable environments. The promising initial results of in vivo screening merit further in-depth exploitation of their PGP properties, as well as the underlying mechanism of PGPR-elicited tolerance.

## Methods

### Sampling sites and sample collection

The sampling sites were selected using as a criterion the adverseness of environmental conditions where plants grow, mostly high salt and water deficient. The rhizosphere of the halophytes and/or drought-tolerant plants was sampled in three distinct collection sites during June–September 2018 and 2019 under the harsh conditions of the Mediterranean summer. These were (i) the National Park of Delta Axios (salinity stress), next to the city of Thessaloniki, Greece; (ii) the peri-urban forest of Seich-Sou (drought stress) in the major area of Thessaloniki; and (iii) Santorini island (drought stress) in the Aegean Sea (Fig. 1; Table S6). The wetland of the National Park of Delta Axios, ranging across 33,800 hectares and with more than 350 species and subspecies of plants in a range of habitats, represents a very diverse ecosystem in Greece. The area includes the estuaries of four rivers and has been included in the Natura 2000 network of European ecological regions with code GR1220002^68^. The forest of Seich-Sou is a peri-urban Mediterranean forest to the north and northeast of Thessaloniki that is characterized by acidic soils. It is mainly covered by pine trees. Dominant plants in the understorey are *Cistus* sp., *Thymus* sp., and *Mentha pulegium*. Finally, on Santorini Island, in the Aegean Sea, the local tomato cultivar is very well-adapted to its volcanic soil. Growing under high light, high temperature and no water conditions, it is considered as drought-tolerant^69^.

Regarding the sampling from the National Park of Delta Axios, the rhizospheres of five individuals from the native plants of *Sarcoccornia* sp., *Atriplex* sp., and *Crithmum* sp. were collected in mid-June 2018, when the average day temperature at the sampling date was 25.7 °C (32.7 °C maximum) and there was a relatively long period (17 days) of no rainfall (climatological data of HAO DEMETER). At the early September 2018, rhizosphere samples from the peri-urban forest of Seich-Sou were also collected from the drought-tolerant aromatic plants *Cistus* sp., *Thymus* sp., and *Mentha pulegium* (five individuals each). At the day of sampling, average temperature was 26.3 °C (31.4 °C maximum), and the air’s relative humidity was 34%, while there was no rainfall for a period of 38 days prior to sampling (climatological data of HAO DEMETER). Tomato samples (eight individuals) were obtained at the beginning of June 2019 from two different sampling sites on the island of Santorini, named Emporio and Vlichada. The average day temperature for the past 30 days prior to sampling was 21.3 °C, with 9.2 mm total rainfall (climatic data of the Hellenic National Meteorological Service). All plant samples were placed in sterile bags and brought back to the lab under sterile and cold conditions within 6 h. In addition, from each site, soil samples were collected, air-dried, ground to pass a 2-mm sieve and analysed for particle size distribution^70^ and chemical properties according to the methods reported by Sparks^71^.

### Isolation of rhizobacteria

To isolate rhizospheric bacteria, the bulk soil was removed with manual shaking of the root, and root material of 0.5–1 g from each plant was transferred into phosphate saline buffer (PBS). A final step of 10-min sonication (Transonic 460, frequency 35 kHz) was applied to release the bacteria in the PBS buffer^72^. Following the serial dilution method, an aliquot of the supernatant was spread on three different nutrient media, i.e. 869 medium prepared as described by Eevers et al.^73^, Nutrient Agar Glycerol (Composition: Βactopeptone 3.3 g L^−1^, Nutrient broth 2.7 g L^−1^, Yeast extract 2 g L^−1^, Glycerole 25 mL L^−1^, BactoAgar 15 g L^−1^) and R2-Α Agar (18 g L^−1^, Sigma Aldrich, USA), and incubated at 27 °C for 24–48 h. After 48 h, single bacterial colonies were isolated based on their macroscopic differences and cultured separately in order to obtain new individual cultures. Isolates were identified based on colony morphology and pigmentation and were stored in glycerol stock at − 80 °C until further analysis.

### Identification of PGP traits of bacterial isolates

Isolates stored at − 80 °C were plated on nutrient medium to be further used as starting material for bioassays. All measurements were carried out in triplicates. The optical density measurements were performed with the Epoch2 microplate spectrophotometer (ΒioTek, USA). All the isolated rhizobacteria were screened for their ability to produce siderophores and auxin. By contrast ACC deaminase activity, P-solubilization and organic acids production were tested over selected rhizobacteria that were found to be positive in the first two bioassays.

For the siderophore production, 10 μL of bacterial culture were plated on Chrome Azurol Sulphonate (CAS) medium^74^. The color change of the substrate from blue to orange and the creation of an orange circle (halo) around single colonies was considered as a positive signal^75^.

The production of IAA was measured by the method described by Patten & Glick^32^ with some modifications. Briefly, bacterial strains were grown aerobically at 27 °C with continuous shaking in rich nutrient broth supplemented with tryptophane. After 24 h, 3 mL from the culture’s supernatant were transferred into 3 mL Salkowski reagent (0.5 M FeCl_3_, 70% perchloric acid). The solution was vortexed and kept at room temperature for 30 min. Development of pink pigmentation was considered as a positive reaction. To measure IAA production, the optical density was recorded at 530 nm, and IAA concentration was determined by comparison with the standard curve of different concentrations of IAA (ICN Pharmaceuticals, Cleveland, Ohio, USA) in the range 0.01–1 mM measured at 530 nm.

The detection of ACC deaminase activity of the isolated bacteria was based on their ability to use ACC deaminase as nitrogen source, according to the protocol described by Penrose & Glick^15^. In brief, bacterial colonies were grown aerobically in rich medium broth for up to 24 h, and after centrifugation, the bacterial pellets were transferred in DF salts minimal medium^76^, and incubated for 48 h. In particular, three different culture conditions were used: DF salts minimal medium with ACC as nitrogen source, DF salts minimal medium with (NH_4_)_2_SO_4_ as nitrogen source (positive control) and DF salts minimal medium without any nitrogen source (negative control). The optical density was measured at 405 nm. As positive control the strain *Pseudomonas putida* UW4 was used^77^.

The quantitative assessment of ACC deaminase activity was done spectrophotometrically in terms of alpha-ketobutyrate production as previously described by Penrose & Glick^15^. Briefly, bacterial cells were washed twice with 0.1 M Τris-HCl (pH 7.6) and were suspended in 600 μL 0.1 Μ Τris-HCl (pH 8.5) and 30 μL toluene. The supernatant was incubated in 0.56 N HCl and 2,4-dinitrophenylhydrazine for 30 min at 27 °C. After the addition of NaOH, positive strains were considered those with a change of colouration from yellow to brown. The quantification of ACC deaminase activity was based on the standard curve of the alpha-ketobutyrate (Sigma Aldrich) measured in a range of concentrations (0.1–1 mM) at 540 nm. All measurements were carried out in triplicates. One unit of ACC deaminase activity was expressed as the amount of alpha-ketobutyrate produced per mg of protein per hour. Bovine serum albumin (Sigma Aldrich) was used as standard for the quantification of protein content of the extracts according to Bradford method^78^.

The bioassay for the solubilization of P was based on the ability of some bacteria to solubilize phosphoric calcium in National Botanical Research Institute’s phosphate growth medium (NBRIP) agar plates79. The bacteria were inoculated with sterile toothpicks. For the positive strains, a halo zone was observed around the colonies after 5–7 days and the solubilization index was expressed as follows: (colony diameter + halo zone diameter)/colony diameter^80^.

To evaluate the production of organic acids, the strains were grown aerobically in rich medium broth at 27 °C for up to 24 h and the supernatant was incubated in sucrose-tryptone medium, as described by Abbamondi et al.^34^. When Alizarine red was added, the change of the solution’s color to yellow was considered as positive signal.

### 16S rRNA gene sequencing of isolated rhizobacteria

Twenty-five (25) rhizobacterial strains with potential PGP traits were further selected to be taxonomically assigned to their closest relatives. The strains were grown aerobically at 27 °C with continuous shaking in rich nutrient broth. PCR amplification of the V3-V4 region of the 16S rRNA gene was performed using the Bakt_341F (5′-CCTACGGGNGGCWGCAG-3′) and Bakt_805R (5′-GACTACHVGGGTATCTAATCC-3′) primers^81^. For the PCR amplification step, 0.5 μL of each liquid bacterial culture was used. PCR included an initial denaturation step at 95 °C for 5 min, which was followed by 35 cycles consisting of denaturation at 95 °C for 30 s, annealing at 50 °C for 30 s, and elongation at 72 °C for 1 min; a final 7 min elongation step at 72 °C was added. The PCR products were visualized on a 1% agarose gel under UV light. All of them gave a positive amplification signal, and the PCR products were purified with the PureLink.

Quick PCR Purification Kit (Thermo Fisher Scientific, Waltham Massachusetts, USA) according to the manufacturer’s instructions. Sequence data of the purified products were obtained by Sanger Sequencing technologies at Eurofins Genomics AT GmbH (Austria). All produced reads of the 16S rRNA gene fragments were compared with the BLAST function against the SILVA v.132 database, containing 1,861,569 curated bacterial SSU rRNA gene sequences^82^, and closest relatives were verified with NCBI database searches.

### Whole genome sequencing of selected PGPR and read processing

Overall, based on their different morphological features, their in vitro PGP-related traits, as well as their 16 s rRNA gene variability, eight rhizobacterial strains were further selected for whole genome sequencing in order to identify loci potentially associated with PGP functional traits. Briefly, DNA was extracted by their respective liquid cultures, with the use of the Macherey–Nagel NucleoSpin Soil Genomic DNA Isolation Kit, according to the manufacturer’s instructions. The concentration and quality of the recovered DNA was checked using the Thermo Scientific NanoDrop spectrophotometer (Thermo Fisher Scientific, Waltham Massachusetts, USA), and was confirmed by gel electrophoresis. Library construction was performed with the use of the Illumina Nextera XT sample prep, index kits and the MiSeq Reagent Micro Kit v2 for the 150 + 150 bp paired end sequencing (Illumina, San Diego, CA, USA), according to the manufacturer’s instructions. Whole genome sequencing was performed on a MiSeq Illumina sequencer at the core facilities of the Institute of Applied Sciences (Centre for Research and Technology, Greece).

The raw reads were first quality screened using the “FastQC quality control” tool of Galaxy v.0.72, and then pre-treated using the “Filter by quality” tool of Galaxy v.0.72^83^, by removing the low-quality reads (with Phred score below 20) and those with < 90% high-quality score distribution. Forward and reverse reads were joined and then assembled into bigger contigs with the software MEGAHIT v.1.0^84^ using a 60 bp overlap and a threshold of minimum 300 bp length in the produced contigs. The contigs were functionally annotated with Prokka^85^. In order to infer the taxonomic affiliation of the eight strains, a phylogenetic tree was constructed based on Multilocus Sequence Analysis (MLSA) of five housekeeping genes (*rpaA* – *rpoA* – *infB* – *gyrB* – *atpD*) (Fig. S1)^16,18^. The sequences of these individual genes of the strains identified by Prokka searches, along with those from reference strains of closest relatives retrieved from the NCBI database, were aligned using ClustalW^86^, and were combined to form a concatenated sequence by BioEdit^87^. MEGA-X software^88^ was used to construct the topological tree using the Neighbor-Joining algorithm with the Maximum Composite Likelihood model. To estimate the level of support for each branch, 1,000 bootstrap samples were generated^89^.

The reference genomes that were closely branched in the produced phylogenetic tree were selected to map the respective eight rhizobacterial genomes of the present study using Geneious Prime 2019.2.3 (https://www.geneious.com). The assembled genomes were screened, and the CDS of each genome were extracted. Functional analysis of the extracted CDS by Cluster of Orthologous Genes (COG) was performed using the WebMGA^90^.

### Stress experiments with PGPR inoculated tomato plants

The next step was to test the selected potential PGPR (AXSa06, AXSa07, SSTh08, SSCi02, SAVSo04, SAESo11, SAESo12, and SAESo14) in pot experiments in the growth chamber. As the first two strains were isolated from saline environmental conditions, they were tested as inoculants under salt stress, and similarly, the rest of the strains isolated from non-irrigated conditions, were tested under drought stress. The tomato variety “ACE55” was used for the experiments. Seeds were surface sterilized for 3 min in 70% ethanol (v/v) solution and subsequently for 2 min in 2.4% (v/v) sodium hypochlorite solution. The efficacy of the seed sterilization protocol was tested by placing 24 h dried seeds on NAG (Nutrient Agar Glycerol) plates and incubated for 24–48 h to observe any bacterial growth. For synchronized growth, the seeds were placed in the dark for 24 h at 4 °C prior to sterilization and then incubated for 72hrs in PNS agar plates (1.8% agar) at 25 °C.

Each strain was inoculated in nutrient glycerol liquid medium and incubated overnight at 27 °C prior to inoculation procedure, and 1.5 ml of the liquid culture was centrifuged (4 °C, 2,200 rpm, 15 min). The pellets of the bacterial strains were collected, and the optical density of the bacterial suspension at 600 nm was adjusted to 0.7 by adding PBS. Selected seeds were then "bacterized" for 30 min in a mixture of the bacterial suspension and 2% methyl cellulose solution (w/v) (Methylcellulose (MC), viscosity 4,000 cp, Sigma (Merck)) in a 1: 1 (v/v) ratio^91^. Control uninoculated seeds were immersed in a mixture of PBS and MC (1:1 (v/v)). Inoculated seeds were then placed in pots filled with a sterilized mixture of peat and perlite (3:1 v/v) in a growth chamber (Snijders Microclima 1,750, Snijders Scientific BV, Netherlands) under non-stressed conditions at 16/8 h and 25/22 °C. After 21 days for all strains (except for AXSa06 which was 14 days), water was suspended for 3 days, and stress treatments were applied. For salt stress, 200 mM NaCl was supplied every second day. To induce drought stress, watering was withheld until the soil volumetric water content decreased to ~ 0.15 m^3^ m^−3^, compared to control plants that had ~ 0.4 m^3^ m^−3^ (ProCheck Decagon Devices, Pullman, WA, USA, equipped with TEROS 10 soil sensor). Each treatment consisted of 20 plants. After 7 days of stress, plant height and total above-the-ground biomass were measured. Duncan’s multiple test was conducted by the statistical package SPSS (version 24) to determine statistical differences between treatments and PGPR at a 5% significance level.

## Supplementary information

Supplementary Information.

## Data Availability

Raw reads were submitted to GenBank-SRA under the Accession Number PRJNA606387.
