# Supplementary material for: Plant growth promoting rhizobacteria isolated from halophytes and drought-tolerant plants: genomic characterisation and exploration of phyto-beneficial traits
Source: Sci Rep. 2020 Sep 9;10:14857. doi: 10.1038/s41598-020-71652-0 (PMC7481233; doi:10.1038/s41598-020-71652-0)
Supplement: Supplementary file 1 — Supplementary Information. [file 41598_2020_71652_MOESM1_ESM.docx]

**Plant-Growth-Promoting Rhizobacteria isolated from halophytes and drought-tolerant plants: Genomic characterisation and exploration of phyto-beneficial traits**

Kleopatra Leontidou^1*^, Savvas Genitsaris^2,3*^, Anastasia Papadopoulou^1^, Natalie Kamou^1^, Irene Bosmali^4^, Theodora Matsi^5^, Panagiotis Madesis^4^, Despoina Vokou^3^, Katerina Karamanoli^1⁑^& Ifigeneia Mellidou^1,6⁑^

* equal contribution

*^1^Laboratory of Agricultural Chemistry, School of Agriculture, Aristotle University of Thessaloniki, 54124 Thessaloniki, Greece*

*^2^International Hellenic University, 57001 Thermi, Greece*

*^3^Department of Ecology, School of Biology, Aristotle University of Thessaloniki, 54124 Thessaloniki, Greece*

*^4^Institute of Applied Biosciences, CERTH, Thessaloniki, 57001, Greece*

*^5^Soil Science Laboratory, School of Agriculture, Aristotle University of Thessaloniki, 54124 Thessaloniki, Greece*

*^6^Institute of Plant Breeding and Genetic Resources, HAO ELGO-DEMETER. Thermi, Thessaloniki, GR-57001, Greece*

^⁑^Corresponding authors: Mellidou Ifigeneia, [imellidou@ipgrb.gr](mailto:imellidou@ipgrb.gr); [ifimellidou@gmail.com](mailto:ifimellidou@gmail.com); Katerina Karamanoli, [katkar@agro.auth.gr](mailto:katkar@agro.auth.gr)

**Supplementary Table** **S1.** Rhizobacterial isolates and their morphological characteristics.

| **Strain** | **Isolated from** | | **Colony morphology (color, size, other)** | **Growth Medium** | **Biochemical characterization** | |
| --- | --- | --- | --- | --- | --- | --- |
|  | **Sampling site** | **Plant species** |  |  | **Siderophore production (Colony diameter in mm)** | **IAA production (μg mL^-1^)** |
| AXCr01 | National Park of Delta Axios | *Crithmum* sp. | Light yellow | 869 | 0 | Nd |
| AXCr02 | National Park of Delta Axios | *Crithmum* sp. | White, big, sticky | 869 | 5 | 35.60 |
| AXCr03 | National Park of Delta Axios | *Crithmum* sp. | White, small | 869 | 5 | Nd |
| AXCr04 | National Park of Delta Axios | *Crithmum* sp. | Light white | 869 | 0 | Nd |
| AXCr05 | National Park of Delta Axios | *Crithmum* sp. | Light red | 869 | 0 | Nd |
| AXCr06 | National Park of Delta Axios | *Crithmum* sp. | Red | 869 | 0 | Nd |
| AXCr07 | National Park of Delta Axios | *Crithmum* sp. | Light red | 869 | 5 | 65.84 |
| AXCr08 | National Park of Delta Axios | *Crithmum* sp. | Orange | 869 | 5 | 58.86 |
| AXCr09 | National Park of Delta Axios | *Crithmum* sp. | Brown | NAG | 0 | Nd |
| AXCr10 | National Park of Delta Axios | *Crithmum* sp. | Light white, big | NAG | 0 | Nd |
| AXCr11 | National Park of Delta Axios | *Crithmum* sp. | Yellow | NAG | 0 | Nd |
| AXCr12 | National Park of Delta Axios | *Crithmum* sp. | White, non-symmetric | NAG | 0 | Nd |
| AXAt01 | National Park of Delta Axios | *Atriplex* sp. | Light yellow | 869 | 0 | 28.62 |
| AXAt02 | National Park of Delta Axios | *Atriplex* sp. | Light red | 869 | 0 | 23.97 |
| AXAt03 | National Park of Delta Axios | *Atriplex* sp. | White, big | 869 | 0 | Nd |
| AXAt04 | National Park of Delta Axios | *Atriplex* sp. | Yellow, big | 869 | 0 | Nd |
| AXAt05 | National Park of Delta Axios | *Atriplex* sp. | With brown halo | NAG | 0 | Nd |
| AXAt06 | National Park of Delta Axios | *Atriplex* sp. | With brown halo | NAG | 0 | Nd |
| AXAt07 | National Park of Delta Axios | *Atriplex* sp. | White, non-symmetric | NAG | 0 | Nd |
| AXAt08 | National Park of Delta Axios | *Atriplex* sp. | Yellow, non-symmetric | NAG | 0 | 105.30 |
| AXSa01 | National Park of Delta Axios | *Sarcocornia* sp. | Orange, small | 869 | 0 | 72.81 |
| AXSa02 | National Park of Delta Axios | *Sarcocornia* sp. | Yellow | 869 | 5 | 44.91 |
| AXSa03 | National Park of Delta Axios | *Sarcocornia* sp. | Red | 869 | 0 | Nd |
| AXSa04 | National Park of Delta Axios | *Sarcocornia* sp. | Red | 869 | 0 | Nd |
| AXSa05 | National Park of Delta Axios | *Sarcocornia* sp. | With brown halo | NAG | 0 | Nd |
| AXSa06 | National Park of Delta Axios | *Sarcocornia* sp. | White, big | NAG | 5 | 31.25 |
| AXSa07 | National Park of Delta Axios | *Sarcocornia* sp. | Yellow, non-symmetric | NAG | 6 | 36.88 |
| AXSa08 | National Park of Delta Axios | *Sarcocornia* sp. | Red | NAG | 0 | 56.53 |
| SSTh01 | Seich-Sou Forest | *Thymus* sp. | Yellow | 869 | 1 | 68.16 |
| SSTh02 | Seich-Sou Forest | *Thymus* sp. | White | 869 | 0 | 23.98 |
| SSTh03 | Seich-Sou Forest | *Thymus* sp. | White | 869 | 9 | 26.88 |
| SSTh04 | Seich-Sou Forest | *Thymus* sp. | Yellow | 869 | 0 | 56.53 |
| SSTh05 | Seich-Sou Forest | *Thymus* sp. | White, round | 869 | 0 | Nd |
| SSTh06 | Seich-Sou Forest | *Thymus* sp. | Yellow | NAG | 8 | 36.67 |
| SSTh07 | Seich-Sou Forest | *Thymus* sp. | Yellow | NAG | 4 | 32.92 |
| SSTh08 | Seich-Sou Forest | *Thymus* sp. | White, transparent | R2 | 1 | 30.00 |
| SSTh09 | Seich-Sou Forest | *Thymus* sp. | Yellow | R2 | 6 | 29.58 |
| SSTh10 | Seich-Sou Forest | *Thymus* sp. | Light white | R2 | 0 | Nd |
| SSTh11 | Seich-Sou Forest | *Thymus* sp. | With biofilm | R2 | 7 | 22.08 |
| SSMe01 | Seich-Sou Forest | *Menta pulegium* | Orange, slimy | 869 | 4 | 17.92 |
| SSMe02 | Seich-Sou Forest | *Menta pulegium* | Yellow | R2 | 5 | 27.29 |
| SSMe03 | Seich-Sou Forest | *Menta pulegium* | Dark orange | R2 | 14 | 34.58 |
| SSMe04 | Seich-Sou Forest | *Menta pulegium* | Orange, light | R2 | 9 | 36.46 |
| SSMe05 | Seich-Sou Forest | *Menta pulegium* | Light white, round | R2 | 0 | Nd |
| SSMe06 | Seich-Sou Forest | *Menta pulegium* | Brown | R2 | 0 | Nd |
| SSMe07 | Seich-Sou Forest | *Menta pulegium* | Orange | R2 | 0 | Nd |
| SSMe08 | Seich-Sou Forest | *Menta pulegium* | Yellow, round | R2 | 0 | 35.60 |
| SSMe09 | Seich-Sou Forest | *Menta pulegium* | Yellow | R2 | 0 | 54.20 |
| SSCi01 | Seich-Sou Forest | *Cistus* sp. | Light yellow, slimy | NAG | 9 | 25.21 |
| SSCi02 | Seich-Sou Forest | *Cistus* sp. | White | NAG | 4 | 112.50 |
| SSCi03 | Seich-Sou Forest | *Cistus* sp. | White | NAG | 0 | Nd |
| SSCi04 | Seich-Sou Forest | *Cistus* sp. | Yellow | NAG | 0 | Nd |
| SSCi05 | Seich-Sou Forest | *Cistus* sp. | Light white, big | R2 | 11 | 22.71 |
| SSCi06 | Seich-Sou Forest | *Cistus* sp. | White, small | R2 | 0 | Νd |
| SSCi07 | Seich-Sou Forest | *Cistus* sp. | Yellow, small | R2 | 0 | Nd |
| SSCi08 | Seich-Sou Forest | *Cistus* sp. | Yellow, fluorescent | R2 | 4 | Nd |
| SSCi09 | Seich-Sou Forest | *Cistus* sp. | Brown, small | R2 | 0 | Nd |
| SSCi10 | Seich-Sou Forest | *Cistus* sp. | White, big | R2 | 0 | Nd |
| SAVSo01 | Vlichada-Santorini | *Solanum lycopersicum* | White, small | 869 | 4 | 37.29 |
| SAVSo02 | Vlichada-Santorini | *Solanum lycopersicum* | White, transparent | 869 | 0 | 51.25 |
| SAVSo03 | Vlichada-Santorini | *Solanum lycopersicum* | Yellow, small | R2 | 0 | 48.75 |
| SAVSo04 | Vlichada-Santorini | *Solanum lycopersicum* | Transparent | R2 | 5 | 43.96 |
| SAVSo05 | Vlichada-Santorini | *Solanum lycopersicum* | Transparent | R2 | 5 | 92.71 |
| SAVSo06 | Vlichada-Santorini | *Solanum lycopersicum* | Dark white, big | R2 | 0 | 64.58 |
| SAESo01 | Emporio-Santorini | *Solanum lycopersicum* | Orange | R2 | 0 | 48.54 |
| SAESo02 | Emporio-Santorini | *Solanum lycopersicum* | Yellow, big | R2 | 0 | 35.42 |
| SAESo03 | Emporio-Santorini | *Solanum lycopersicum* | White, transparent | R2 | 0 | 52.71 |
| SAESo04 | Emporio-Santorini | *Solanum lycopersicum* | Yellow, small | R2 | 0 | 43.96 |
| SAESo05 | Emporio-Santorini | *Solanum lycopersicum* | White, big | R2 | 0 | 92.71 |
| SAESo06 | Emporio-Santorini | *Solanum lycopersicum* | White, transparent | R2 | 0 | 54.17 |
| SAESo07 | Emporio-Santorini | *Solanum lycopersicum* | Yellow, dark | R2 | 6 | 43.96 |
| SAESo08 | Emporio-Santorini | *Solanum lycopersicum* | White, big | R2 | 0 | 39.17 |
| SAESo09 | Emporio-Santorini | *Solanum lycopersicum* | White | R2 | 0 | 40.63 |
| SAESo10 | Emporio-Santorini | *Solanum lycopersicum* | White, slimy | R2 | 0 | 39.79 |
| SAESo11 | Emporio-Santorini | *Solanum lycopersicum* | Yellow, slimy | NAG | 5 | 90.42 |
| SAESo12 | Emporio-Santorini | *Solanum lycopersicum* | White, slimy | NAG | 6 | 105.2 |
| SAESo13 | Emporio-Santorini | *Solanum lycopersicum* | White | NAG | 6 | 55.42 |
| SAESo14 | Emporio-Santorini | *Solanum lycopersicum* | Orange | 869 | 5 | 74.38 |
| SAESo15 | Emporio-Santorini | *Solanum lycopersicum* | Dark yellow | 869 | 6 | 47.29 |
| SAESo16 | Emporio-Santorini | *Solanum lycopersicum* | White, transparent | 869 | 5 | 38.75 |
| SAESo17 | Emporio-Santorini | *Solanum lycopersicum* | White, non-symmetric | 869 | 0 | 47.29 |
| SAESo18 | Emporio-Santorini | *Solanum lycopersicum* | Yellow | 869 | 0 | 45.42 |
| SAESo19 | Emporio-Santorini | *Solanum lycopersicum* | White, transparent | 869 | 6 | 36.25 |
| SAESo20 | Emporio-Santorini | *Solanum lycopersicum* | Orange | 869 | 0 | 54.58 |
| SAESo21 | Emporio-Santorini | *Solanum lycopersicum* | Orange, transparent | 869 | 0 | 47.29 |

Nd: Not detectable

**Supplementary Table S2.** The strains which were selected as potential Plant-Growth-Promoting-Rhizobacteria (PGPR), their putative high-level taxonomic affiliation based on SILVA database blastn searches, their closest relative and the isolation source of the closest relative.

| **Strain** | **Putative High-Level Taxonomic Affiliation** | **Closest relative (% similarity)**  **[NCBI accession number]** | **Isolation Source of Closest Relative** |
| --- | --- | --- | --- |
| AXSa06 | Firmicutes | *Bacillus megaterium* (97%) [KC246046] | Soil of ramie plantation |
| AXSa07 | Gammaproteobacteria | *Pseudomonas* sp. (98%) [KC117524] | Ginseng field |
| SSMe01 | Bacteroidetes | *Pedobacter rhizosphaerae* (96%) [AM279214] | Rhizosphere soil |
| SSMe02 | Gammaproteobacteria | *Pseudomonas* sp. (97%) [LMQZ01000006] | Leaf of *Arabidopsis thaliana* |
| SSMe03 | Firmicutes | *Bacillus* *cereus* (96%) [FMJJ01000012] | Culture strain |
| SSMe04 | Gammaproteobacteria | *Pseudomonas* sp. (97%) [KU569690] | Rhizosphere of *Crocus sativus* |
| SSCi01 | Gammaproteobacteria | *Pseudomonas* sp. (97%) [LMQZ01000006] | Leaf of *Arabidopsis thaliana* |
| SSCi02 | Gammaproteobacteria | *Pantoea* sp. (98%) [JX279933] | Saffron rhizosphere |
| SSCi05 | Gammaproteobacteria | *Luteibacter* sp. (94%) [KT005672] | Forest soil |
| SSTh03 | Gammaproteobacteria | *Pseudomonas* sp. (97%) [KU569690] | Rhizosphere of *Crocus sativus* |
| SSTh06 | Gammaproteobacteria | *Pseudomonas* sp. (98%) [KU569690] | Rhizosphere of *Crocus sativus* |
| SSTh07 | Gammaproteobacteria | *Pseudomonas* sp. (98%) [HF952550] | Rhizosphere soil |
| SSTh08 | Gammaproteobacteria | *Pseudomonas* sp. (98%) [KU569690] | Rhizosphere of *Crocus sativus* |
| SSTh09 | Gammaproteobacteria | *Pseudomonas* sp. (98%) [KU569690] | Rhizosphere of *Crocus sativus* |
| SSTh11 | Gammaproteobacteria | *Pseudomonas* sp. (97%) [LMQZ01000006] | Leaf of *Arabidopsis thaliana* |
| SAVSo01 | Firmicutes | *Bacillus cereus* (98%) [HQ285924] | Mycorrhiza growing on tomato |
| SAVSo04 | Gammaproteobacteria | *Acinetobacter calcoaceticus* (95%) [MN429316] | Entomopathogenic nematodes |
| SAVSo06 | Gammaproteobacteria | *Lysobacter* sp. (81%) [KC492987] | *Pinus patula* rhizosphere |
| SAESo05 | Gammaproteobacteria | *Enterobacter* sp. (96%) [HQ246257] | Tilapia fish culture system |
| SAESo06 | Gammaproteobacteria | *Pseudomonas* sp. (81%) [AY748893] | Rhizosphere of *Nicotiana glauca* |
| SAESo11 | Firmicutes | *Bacillus cereus* (96%) [KU752874] | Crude oil contaminated soil |
| SAESo12 | Unidentified Bacterium | Unidentified Sequence | - |
| SAESo14 | Bacteroidetes | *Chryseobacterium* sp. (93%) [KJ482840] | Soil |
| SAESo15 | Firmicutes | *Bacillus mycoides* (97%) [AB819834] | Mangrove sediment |
| SAESo19 | Gammaproteobacteria | *Pseudomonas* sp. (85%) [KY438733] | Animal foods |

**Supplementary Table S3.** General features of the assembled contigs after whole genome sequencing of the eight rhizobacterial strains that were selected for their Plant-Growth Promoting properties, based on Prokka functional annotations.

| **Analysis Statistics** | **Strains** | | | | | | | |
| --- | --- | --- | --- | --- | --- | --- | --- | --- |
|  | **AXSa06** | **AXSa07** | **SSTh08** | **SSCi02** | **SAVSo04** | **SAESo11** | **SAESo12** | **SAESo14** |
| Size (bp) | 5,106,071 | 5,431,420 | 6,736,907 | 4,916,956 | 3,777,101 | 6,516,086 | 6,569,598 | 5,122,873 |
| Number of contigs | 563 | 557 | 1,873 | 518 | 1,522 | 1,522 | 4,429 | 1,653 |
| Mean contig length | 9,069 | 9,751 | 3,597 | 9,492 | 2,482 | 4,281 | 1,483 | 3,099 |
| Mean GC content (%) | 66 ± 5 % | 64 ± 5 % | 62 ± 4 % | 55 ± 5 % | 39 ± 5 % | 61 ± 5 % | 64 ± 4 % | 38 ± 10 % |
| Predicted protein features | 3,487 | 3,741 | 5,430 | 3,625 | 3,513 | 4,950 | 7,794 | 4,068 |
| Predicted rRNA features | 53 | 44 | 40 | 47 | 57 | 50 | 66 | 46 |

**Supplementary Table S4.** Functional cluster of orthologous genes (COG) classification (number of annotated CDS) of the eight rhizobacterial strains that were selected for their Plant-Growth Promoting properties.

| **COG functional class** | **Number of annotated CDS** | | | | | | | | | | | | | | | | |
| --- | --- | --- | --- | --- | --- | --- | --- | --- | --- | --- | --- | --- | --- | --- | --- | --- | --- |
|  | **AXSa06** | | **AXSa07** | | | **SSTh08** | | **SSCi02** | | **SAVSo04** | | **SAESo11** | | **SAESo12** | | **SAESo14** | |
|  | *Pseudomons oryzihabitans* | | *Pseudomonas oryzihabitans* | | | *Pseudomonas putida* | | *Pantoea brenneri* | | *Acinetobacter calcoaceticus* | | *Pseudomonas putida* | | *Pseudomonas putida* | | *Chryseobacterium* sp. | |
| **Metabolism** |  | |  | |  |  |  |  |  |  |  |  |  |  |  |  |  |
| Energy production and conversion | 127 | (5.86) | 130 | (5.89) | | 137 | (5.93) | 124 | (5.55) | 109 | (6.44) | 137 | (5.84) | 133 | (6.08) | 91 | (5.78) |
| Amino acid transport and metabolism | 189 | (8.73) | 191 | (8.66) | | 196 | (8.46) | 190 | (8.50) | 152 | (8.98) | 199 | (8.48) | 190 | (8.69) | 145 | (9.22) |
| Nucleotide transport and metabolism | 71 | (3.28) | 71 | (3.23) | | 62 | (2.68) | 70 | (3.12) | 60 | (3.54) | 63 | (2.69) | 62 | (2.85) | 56 | (3.57) |
| Carbohydrate transport and metabolism | 109 | (5.03) | 107 | (4.86) | | 106 | (4.59) | 151 | (6.75) | 49 | (2.89) | 106 | (4.53) | 95 | (4.34) | 92 | (5.85) |
| Coenzyme transport and metabolism | 118 | (5.45) | 120 | (5.44) | | 122 | (5.28) | 119 | (5.32) | 94 | (5.55) | 119 | (5.08) | 118 | (5.39) | 89 | (5.66) |
| Lipid transport and metabolism | 61 | (2.82) | 59 | (2.68) | | 62 | (2.68) | 58 | (2.60) | 55 | (3.25) | 65 | (2.78) | 59 | (2.69) | 45 | (2.86) |
| Inorganic ion transport and metabolism | 122 | (5.63) | 128 | (5.81) | | 134 | (5.80) | 137 | (6.12) | 101 | (5.97) | 133 | (5.68) | 130 | (5.95) | 84 | (5.34) |
| Secondary metabolites biosynthesis, transport and catabolism | 35 | (1.63) | 38 | (1.73) | | 44 | (1.90) | 33 | (1.47) | 33 | (1.95) | 45 | (1.92) | 44 | (2.02) | 31 | (1.97) |
| **Cellular processes and signaling** |  |  |  |  | |  |  |  |  |  |  |  |  |  |  |  |  |
| Cell cycle control, cell division, chromosome partitioning | 27 | (1.26) | 27 | (1.23) | | 31 | (1.34) | 29 | (1.30) | 20 | (1.18) | 31 | (1.33) | 28 | (1.28) | 17 | (1.08) |
| Cell wall/membrane/envelope biogenesis | 119 | (5.49) | 117 | (5.30) | | 123 | (5.32) | 134 | (6.00) | 101 | (5.97) | 124 | (5.29) | 116 | (5.29) | 99 | (6.29) |
| Cell motility | 66 | (3.06) | 68 | (3.08) | | 69 | (2.99) | 53 | (2.38) | 36 | (2.13) | 71 | (3.03) | 62 | (2.84) | 4 | (0.25) |
| Post-translational modification, protein turnover, chaperones | 95 | (4.39) | 93 | (4.21) | | 102 | (4.41) | 89 | (3.99) | 70 | (4.13) | 100 | (4.26) | 98 | (4.47) | 63 | (4.01) |
| Signal transduction mechanisms | 87 | (4.03) | 82 | (3.71) | | 79 | (3.42) | 74 | (3.31) | 47 | (2.77) | 78 | (3.31) | 78 | (3.56) | 33 | (2.11) |
| Defense mechanisms | 13 | (0.60) | 18 | (0.82) | | 17 | (0.74) | 18 | (0.81) | 13 | (0.77) | 17 | (0.72) | 18 | (0.82) | 20 | (1.27) |
| Intracellular trafficking, secretion and vesicular transport | 65 | (3.00) | 75 | (3.40) | | 82 | (3.56) | 56 | (2.52) | 57 | (3.37) | 85 | (3.62) | 70 | (3.19) | 27 | (1.72) |
| **Information storage and processing** |  |  |  |  | |  |  |  |  |  |  |  |  |  |  |  |  |
| RNA processing and modification | 1 | (0.05) | 1 | (0.05) | | 1 | (0.04) | 1 | (0.04) | 1 | (0.06) | 1 | (0.04) | 1 | (0.05) | 0 | (0) |
| Translation, ribosomal structure and biogenesis | 139 | (6.43) | 142 | (6.43) | | 138 | (5.98) | 138 | (6.17) | 137 | (8.09) | 140 | (5.97) | 136 | (6.20) | 124 | (7.88) |
| Transcription | 80 | (3.69) | 79 | (3.58) | | 90 | (3.89) | 91 | (4.07) | 61 | (3.60) | 89 | (3.79) | 84 | (3.83) | 61 | (3.88) |
| Replication, recombination and repair | 101 | (4.66) | 108 | (4.89) | | 101 | (4.37) | 103 | (4.61) | 77 | (4.55) | 99 | (4.22) | 98 | (4.47) | 92 | (5.85) |
| **Poorly characterized** |  |  |  |  | |  |  |  |  |  |  |  |  |  |  |  |  |
| General function prediction only | 249 | (11.52) | 250 | (11.32) | | 271 | (11.73) | 258 | (11.54) | 202 | (11.93) | 288 | (12.29) | 253 | (11.53) | 196 | (12.45) |
| Function unknown | 290 | (13.39) | 302 | (13.68) | | 344 | (14.89) | 309 | (13.83) | 218 | (12.88) | 355 | (15.13) | 317 | (14.46) | 204 | (12.96) |
| **Total** | **2167** | | **2208** | | | **2311** | | **2235** | | **1693** | | **2346** | | **2192** | | **1574** | |

**Supplementary Table S5.** The Plant-Growth-Promoting (PGP) traits, the respective genes that are identified as possibly coding for the PGP properties of rhizobacteria, and their number of gene copies of the eight rhizobacterial strains. IAA, Indole-3-acetic acid; ACC, 1-aminocyclopropane-1-carboxylate.

| **PGP Trait** | **Gene** | **AXSa06** | **AXSa07** | **SSTh08** | **SSCi02** | **SAVSo04** | **SAESo11** | **SAESo12** | **SAESo14** |
| --- | --- | --- | --- | --- | --- | --- | --- | --- | --- |
| Phosphate solubilization | Pyrroloquinoline quinone (*pqq*) | 0 | 1 | 0 | 0 | 1 | 0 | 1 | 0 |
|  | Glucose 1-dehydrogenase (*gdh*) | 0 | 0 | 0 | 1 | 2 | 1 | 1 | 0 |
| Nitrogen fixing | Nitrogenases | 0 | 0 | 0 | 0 | 0 | 0 | 0 | 0 |
| Nitric oxide synthesis | Copper-containing nitrite reductase | 0 | 0 | 0 | 0 | 0 | 0 | 0 | 0 |
| IAA biosynthesis and spermidine-related production | Indole-3-pyruvate decarboxylase (*ipdc*) | 0 | 0 | 0 | 0 | 1 | 0 | 0 | 0 |
|  | Tryptophan 2-monooxygenase | 0 | 0 | 0 | 0 | 0 | 0 | 0 | 0 |
|  | Tryptophan synthase (subunit a and b) | 1 | 1 | 2 | 1 | 0 | 2 | 2 | 1 |
|  | Tryptophan aminotransferase | 0 | 0 | 0 | 0 | 0 | 0 | 0 | 0 |
|  | Tryptophan decarboxylase | 0 | 0 | 0 | 0 | 0 | 0 | 0 | 0 |
|  | Indole-3-acetamide hydrolase | 0 | 0 | 0 | 0 | 0 | 0 | 0 | 0 |
|  | Arginine decarboxylase (*speA*) | 0 | 1 | 1 | 0 | 0 | 0 | 1 | 1 |
|  | Agmatine ureohydrolase (*speB*) | 0 | 0 | 0 | 0 | 0 | 0 | 0 | 0 |
|  | Ornithine decarboxylase (*speC*) | 1 | 0 | 1 | 1 | 0 | 1 | 0 | 0 |
|  | SAM decarboxylase (*speD*) | 1 | 0 | 0 | 0 | 0 | 0 | 0 | 0 |
|  | Spermidine synthase (*speE*) | 2 | 1 | 0 | 1 | 0 | 1 | 1 | 0 |
| ACC deaminase activity | ACC deaminase (*acdS*) | 2 | 1 | 0 | 1 | 0 | 1 | 1 | 0 |
|  | D-cysteine desulfhydrase | 1 | 1 | 1 | 1 | 1 | 1 | 0 | 0 |
| Antioxidant activity | Peroxidases (*pox*) | 1 | 0 | 0 | 2 | 0 | 2 | 1 | 0 |
|  | Catalases (*cat*) | 3 | 2 | 1 | 4 | 1 | 4 | 2 | 2 |
|  | Superoxide dismutase (*sod*) | 0 | 1 | 0 | 1 | 2 | 1 | 1 | 3 |
|  | Glutathione peroxidase (*gpx*) | 2 | 2 | 2 | 3 | 0 | 3 | 3 | 1 |
|  | Glutathione reductase (*gr*) | 0 | 1 | 0 | 0 | 1 | 0 | 0 | 0 |
|  | Glutathione S-transferase (*gst*) | 15 | 7 | 3 | 11 | 4 | 11 | 10 | 0 |
| Cell wall and starch degrading | β-glucosidase | 1 | 1 | 1 | 0 | 0 | 0 | 1 | 1 |
|  | α-glucosidase | 0 | 0 | 1 | 0 | 0 | 0 | 0 | 0 |
|  | Endo-1,4-β-xylanase | 0 | 1 | 0 | 0 | 0 | 0 | 0 | 0 |
|  | Glucoamylase | 1 | 0 | 1 | 1 | 0 | 1 | 0 | 0 |
|  | α-amylase | 0 | 0 | 1 | 1 | 0 | 1 | 0 | 2 |
| Choline dehydrogenase | 1 | 1 | 0 | 2 | 1 | 2 | 1 | 0 |  |
|  | Betaine-aldehyde dehydrogenase | 1 | 1 | 1 | 3 | 3 | 3 | 2 | 0 |
|  | Proline dehydrogenase | 0 | 0 | 0 | 0 | 0 | 0 | 0 | 2 |

**Supplementary Table S6.** Informative data about the sampling sites of the study areas and of the plant species, from which the rhizobacteria were isolated.

| **Sampling Sites** | **Latitude (N)** | **Longitude (E)** | **Plant Species** | **Number of Individuals** |
| --- | --- | --- | --- | --- |
|  |  |  |  |  |
| National Park of Delta Axios (AX) | 40°31’19’’ | 22°39’02’’ | *Sarcoccornia* sp. | 5 |
|  |  |  | *Atriplex* sp. | 5 |
|  |  |  | *Crithmum* sp. | 5 |
| Seich-Sou Forest (SS) | 40°37’41’’ | 22°58’15’’ | *Cistus* sp. | 5 |
|  |  |  | *Thymus* sp. | 5 |
|  |  |  | *Mentha pulegium* | 5 |
| Santorini - Emporio (SAE) | 36°20’42’’ | 25°26’44’’ | *Solanum lycopersicum*, cv 'Santorini' (landrace) | 8 |
| Santorini - Vlichada (SAV) | 36°20’57’’ | 25°25’51’’ |  | 8 |


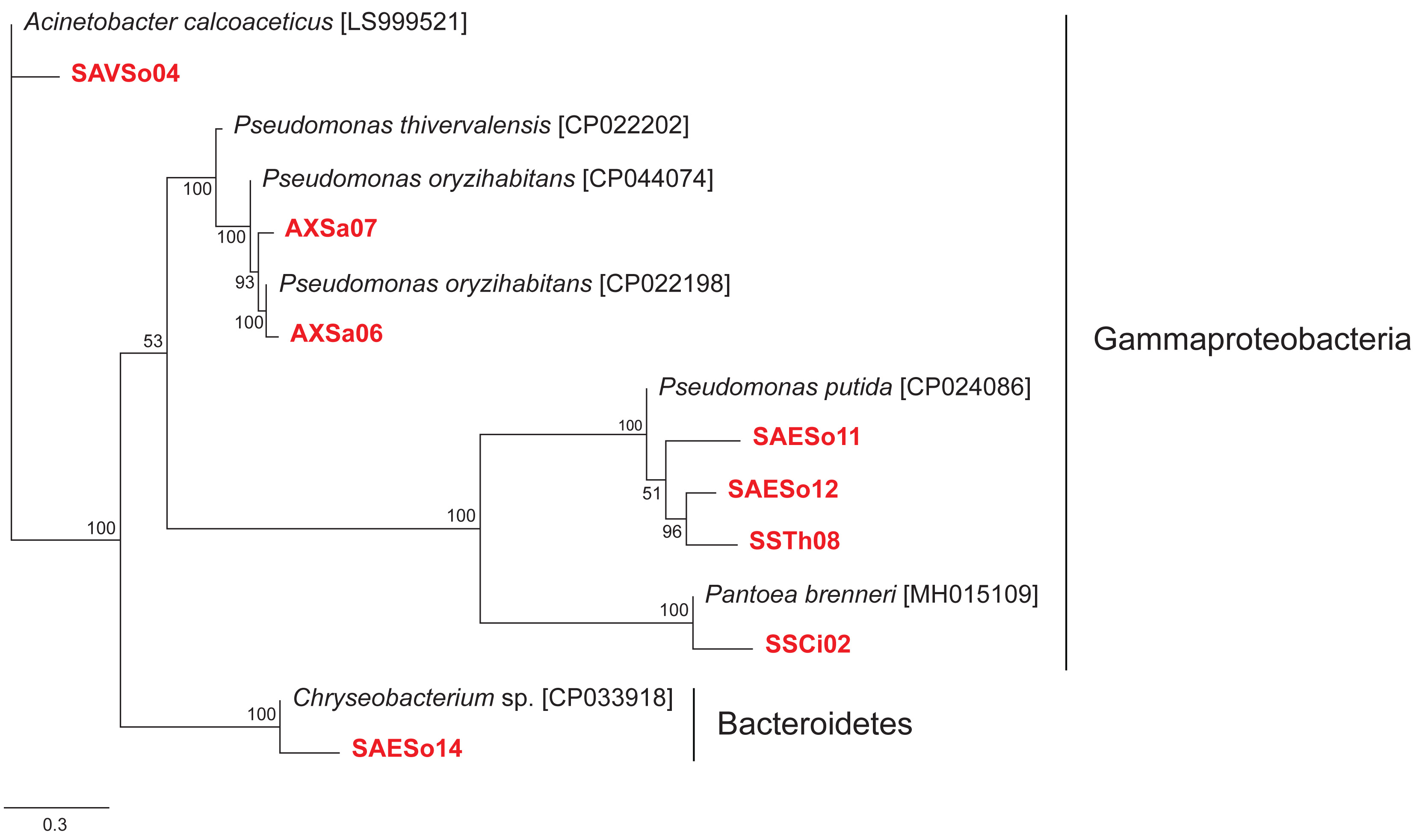


**Supplementary Figure S1.** Phylogenetic tree based on Multilocus Sequence Analysis (MLSA) of five housekeeping genes (rpaA – rpoA – infB – gyrB – atpD) mined from the whole genomes of the eight rhizobacterial strains that were selected for their Plant-Growth-Promoting potential. The evolutionary history was inferred using the Neighbor-Joining method. The percentage of replicate trees in which the associated taxa clustered together in the bootstrap test (1000 replicates) are shown next to the branches when > 50 %. The tree is drawn to scale, with branch lengths in the same units as those of the evolutionary distances used to infer the phylogenetic tree. The evolutionary distances were computed using the Maximum Composite Likelihood method and are in the units of the number of base substitutions per site. All ambiguous positions were removed for each sequence pair (pairwise deletion option). Evolutionary analyses were conducted in the MEGA-X 10.1 software (Kumar et al., 2018; https://www.megasoftware.net/).

Kumar S, Stecher G, Li M, Knyaz C, and Tamura K (2018) MEGA X: Molecular Evolutionary Genetics Analysis across computing platforms. Molecular Biology and Evolution 35:1547-1549.


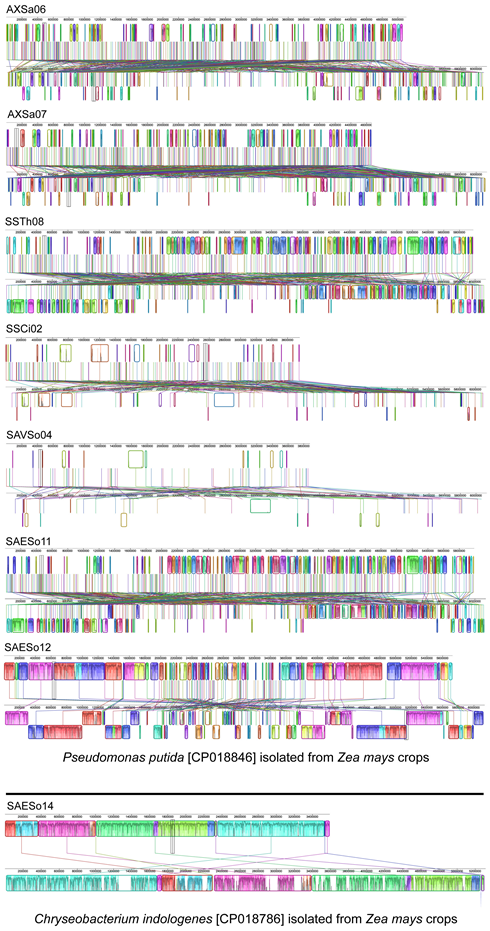


**Supplementary Figure S2.** MAUVE alignments (Darling et al., 2004) of the genomes of the eight rhizobacterial strains that were selected for their Plant-Growth-Promoting potential, with known genomes of rhizobacterial strains isolated from *Zea mays* crops. In particular, for the seven strains that were affiliated to Gammaproteobacteria, a *Pseudomonas putida* strain was selected for the pairwise comparisons (NCBI accession number: CP018846; Niu et al. 2017) and for the strain that was affiliated to Bacteroidetes, a *Chryseobacterium indologenes* strain was selected (NCBI accession number: CP018786; Niu et al. 2017). The coloured blocks represent Locally Collinear Blocks (LCBs), i.e. homologous regions between the genomes that are internally free from genomic rearrangement. LCB weight was determined to > 1564, which has been found to exclude most spurious small matches in genomes of 5Mb sizes (Messeguer & Perna, 2007).

Darling, AC et al. (2004) Mauve: multiple alignment of conserved genomic sequence with rearrangements. Genome Res. 14 1394-403

Messeguer, X, Perna, NT (2007) Analyzing patterns of microbial evolution using the mauve genome alignment system. Methods Mol. Biol. 396 135-152

Niu, B, Paulson, JN, Zheng, X, Kolter, R (2017) Simplified and representative bacterial community of maize roots. PNAS 114 kE2450-2459
